# Liver-Enriched Gene 1, a Glycosylated Secretory Protein, Binds to FGFR and Mediates an Anti-stress Pathway to Protect Liver Development in Zebrafish

**DOI:** 10.1371/journal.pgen.1005881

**Published:** 2016-02-22

**Authors:** Minjie Hu, Yun Bai, Chunxia Zhang, Feng Liu, Zongbin Cui, Jun Chen, Jinrong Peng

**Affiliations:** 1 MOE Key Laboratory for Molecular Animal Nutrition, College of Animal Sciences, Zhejiang University, Hangzhou, China; 2 Institute of Zoology, Chinese Academy of Sciences, Beijing, China; 3 Institute of Hydrobiology, Chinese Academy of Sciences, Wuhan, China; 4 College of Life Sciences, Zhejiang University, Hangzhou, China; University of Pennsylvania School of Medicine, UNITED STATES

## Abstract

Unlike mammals and birds, teleost fish undergo external embryogenesis, and therefore their embryos are constantly challenged by stresses from their living environment. These stresses, when becoming too harsh, will cause arrest of cell proliferation, abnormal cell death or senescence. Such organisms have to evolve a sophisticated anti-stress mechanism to protect the process of embryogenesis/organogenesis. However, very few signaling molecule(s) mediating such activity have been identified. *liver-enriched gene 1* (*leg1*) is an uncharacterized gene that encodes a novel secretory protein containing a single domain DUF781 (domain of unknown function 781) that is well conserved in vertebrates. In the zebrafish genome, there are two copies of *leg1*, namely *leg1a* and *leg1b*. *leg1a* and *leg1b* are closely linked on chromosome 20 and share high homology, but are differentially expressed. In this report, we generated two *leg1a* mutant alleles using the TALEN technique, then characterized liver development in the mutants. We show that a *leg1a* mutant exhibits a stress-dependent small liver phenotype that can be prevented by chemicals blocking the production of reactive oxygen species. Further studies reveal that Leg1a binds to FGFR3 and mediates a novel anti-stress pathway to protect liver development through enhancing Erk activity. More importantly, we show that the binding of Leg1a to FGFR relies on the glycosylation at the 70^th^ asparagine (Asn^70^ or N^70^), and mutating the Asn^70^ to Ala^70^ compromised Leg1’s function in liver development. Therefore, Leg1 plays a unique role in protecting liver development under different stress conditions by serving as a secreted signaling molecule/modulator.

## Author Summary

Although being challenged by stresses from their living environment during embryogenesis, teleost fish harbor a robust genetic program dictating liver development as long as any environmental change, including temperature or natural UV irradiation, is not detrimental. It is therefore of interest to explore the mechanism(s) behind this phenomenon. We showed that Liver-enriched gene 1 (Leg1) plays a unique role in protecting liver development under different stress conditions by serving as a secretory signaling molecule/modulator that binds to FGF receptor and activates the Erk signaling pathway. This finding may explain the adaption of teleost fish in coping with environmental changes.

## Introduction

The process of liver development includes 1) the specification of hepatoblasts from the endoderm, 2) proliferation of hepatoblasts to form the liver primordium (liver bud), and 3) differentiation and proliferation of hepatocytes to form the embryonic liver [1–5]. Liver organogenesis is not only controlled by intrinsic transcription factors such as FoxA factors [6], GATA factors [7], Hhex[8] and Prox1 [9] but also by secreted signaling molecules [10] including FGF [11], BMP [12], Wnt[13] and RA [14] produced by neighboring mesodermal cells/tissues. Strikingly, studies of mouse, chick/quail, *Xenopus* and zebrafish have shown that the molecular events controlling liver development are robustly conserved across these different species although evolutionally, these species are distantly related, especially when considering the obvious anatomic differences in organ initiation and patterning [1–4] and the differences in the circumstances of their embryogenesis. Unlike mammals and birds, teleost fish complete the process of embryogenesis externally. To cope with the stresses brought about by environmental changes, teleost fish have to evolve anti-stress mechanism(s) to protect the process of embryogenesis/organogenesis. However, the molecule(s) mediating such activity is(are) currently unknown. Therefore, it is of great interest to determine whether other such factors, in addition to the aforementioned common factors, protect liver development during external embryogenesis in teleost fish.

*leg1* (*liver enriched gene 1*) is an evolutionally conserved gene in vertebrates that encodes a novel secreted protein Leg1, which contains only a domain of unknown function 781 (DUF781) [15–17]. In zebrafish, there are two copies of the *leg1* gene, namely *leg1a* and *leg1b*, which are closely linked on chromosome 20 [15]. Previous reports showed that knockdown of total Leg1 results in defective liver development, and the expression of *leg1* is modulated by hypoxia conditions [18]. On the basis of the detailed analysis of *leg1a* mutants generated by the TALEN method, we provide strong evidence to demonstrate that Leg1 functions probably as a novel signaling molecule/modulator to protect liver development through Erk phosphorylation under stress conditions, and glycosylation at N^70^ in Leg1a is essential for this function.

## Results

### Loss-of-function of Leg1aconfers a small liver phenotype under different stress conditions

**Fig 1:**
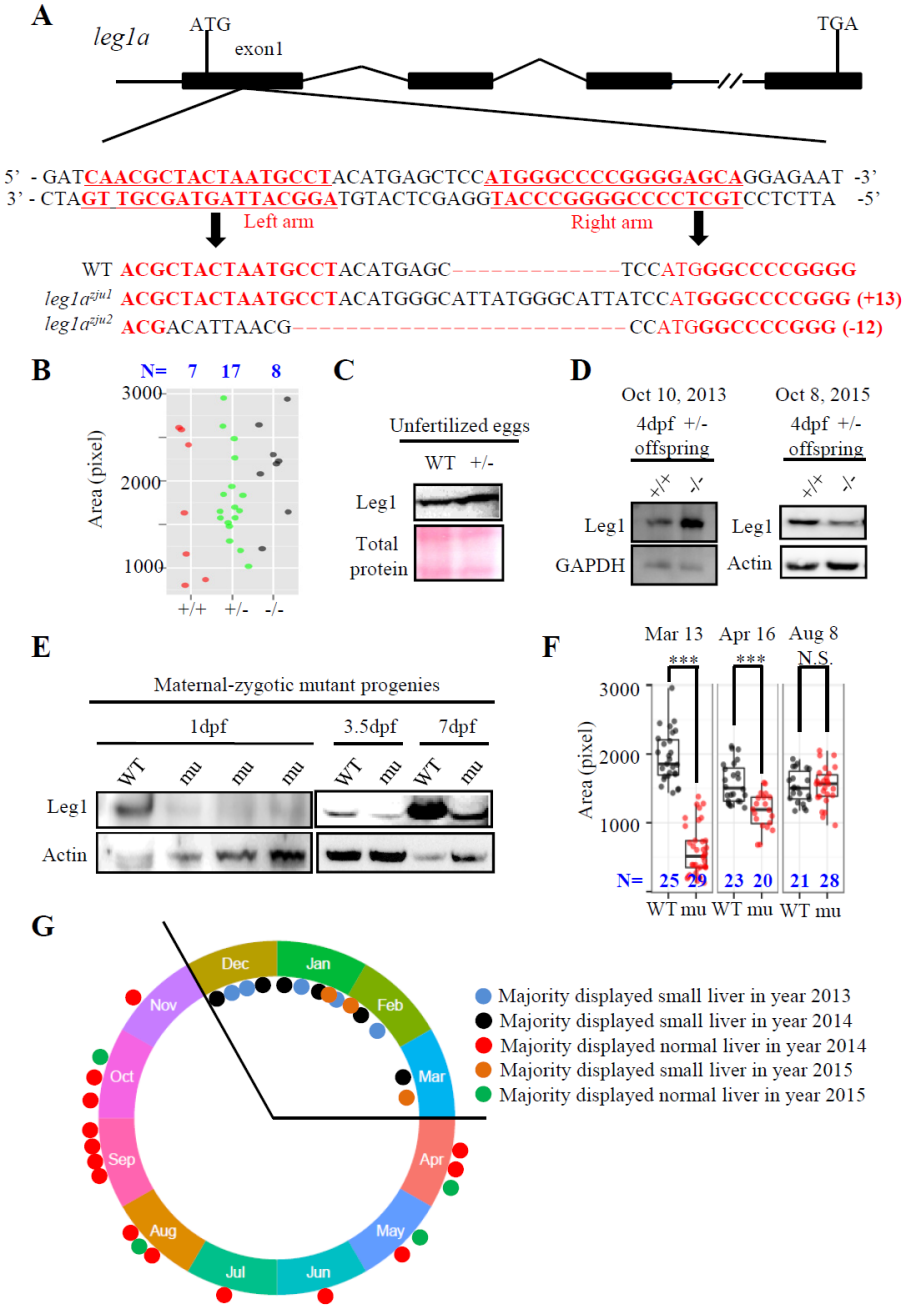
Liver development in the maternal-zygotic *leg1a*^*zju1*^ mutant is amenable to the environmental changes. (A) Top panel: Schematic diagram showing the genomic structure of the *leg1a* gene. Black box: exons; solid line: introns; double slashes: omitted genomic region. Middle panel: The left and right TALEN targeting sequences in the exon1 are lettered in red. Bottom panel: Comparison of the genomic DNA sequence among WT, *leg1a*^*zju1*^ (with 13 bp insertion) and *leg1a*^*zju2*^ (with 12 bp deletion) two mutant alleles. TALEN target sequences are in red. (B-D) The *leg1a*^*zju1*^ homozygotes (-/-) obtained from a cross between heterozygous (+/-) parents showed normal liver development (B). Each dot represents the liver size (measured based on the signal area of *fabp10a*) of a single embryo. Three independent experiments were carried and a representative one is shown here. Total Leg1 was detected in unfertilized eggs (C) and in two independent samples of 4-dpf WT (+/+) and mutant (-/-) embryos collected at different dates (D). (E-G) The maternal-zygotic *leg1a*^*zju1*^ homozygotes (mu) obtained from a cross between homozygous parents lacked Leg1 at 1 dpf, but expressed Leg1b at 3.5 dpf and 7 dpf (E), and showed a small liver phenotype on March 13, an intermediate-sized liver on April 16, and a normal sized liver on August 8, 2015. (F). Recordings of liver phenotype in 32 cases from December 30 2013 to October 4 2015 showed majority of the maternal-zygotic *leg1a*^*zju1*^ homozygotes (mu) exhibited a small liver in 14 cases recorded in cold seasons but a big variation in 18 cases recorded in warm/hot seasons (G). ***, p<0.001, N.S., not significant. Western blot was repeated three times for (C), five times for (D) and (E).

We reported previously that *leg1a* but not *leg1b* was the predominant form expressed during the embryonic stage in zebrafish and that knockdown of *leg1a* resulted in a small liver phenotype [15]. To unequivocally prove the role of *leg1a* in liver development, we generated two *leg1a* mutant alleles, one with a 13-bp insertion (*leg1a*^*zju1*^) and another with a 12-bp deletion (*leg1a*^*zju2*^) (*leg1b* is intact in these two *leg1a* alleles), via the TALEN technique[19] by targeting exon 1 of *leg1a* (Fig 1A). To our surprise, unlike the *leg1a* morphants[15], the *leg1a* homozygous mutant obtained from a cross between either *leg1a*^*zju1/+*^ or *leg1a*^*zju2/+*^ heterozygous male and female did not show an obvious small liver phenotype at 3.5 days post fertilization (dpf) when examined with a liver-specific molecular marker *fatty acid binding protein 10a* (*fabp10a*) (Fig 1B), and both *leg1a*^*zju1*^ and *leg1a*^*zju2*^ homozygous mutants could grow to adulthood and were fertile. We examined the total Leg1 levels by western blot analysis and found no drastic difference between unfertilized eggs from wild-type (WT) and *leg1a*^*zju1/+*^ heterozygous females (Fig 1C). In fact, the *leg1a*^*zju1*^ homozygous mutant obtained from *leg1a*^*zju1/+*^ crosses retained, though showing variations, a considerable level of Leg1 at 4 dpf(Fig 1D), suggesting that maternal Leg1 compensated for the need for Leg1a during early hepatogenesis[20]. The *leg1a*^*zju1*^ homozygous mutant was propagated and allowed to produce progenies. We determined that such *leg1a*^*zju1*^ homozygous progenies (maternal-zygotic mutants) lacked Leg1 (Fig 1E) at 1 dpf but started to express the Leg1b homolog at 3.5 and 7 dpf. Surprisingly, whole-mount *in situ* hybridization (WISH) using the *fabp10a* probe revealed that the maternal-zygotic mutant exhibited a small liver phenotype in a season-dependent manner (Fig 1F, S1A Fig). For example, majority of the maternal-zygotic mutants exhibited a small liver phenotype in 14 cases recorded during the cold season whereas the mutant liver showed a great variation in sizes ranging from normal to small in 18 cases recorded during the warm/hot seasons (Fig 1G, S1B and S1C Fig).

**Fig 2:**
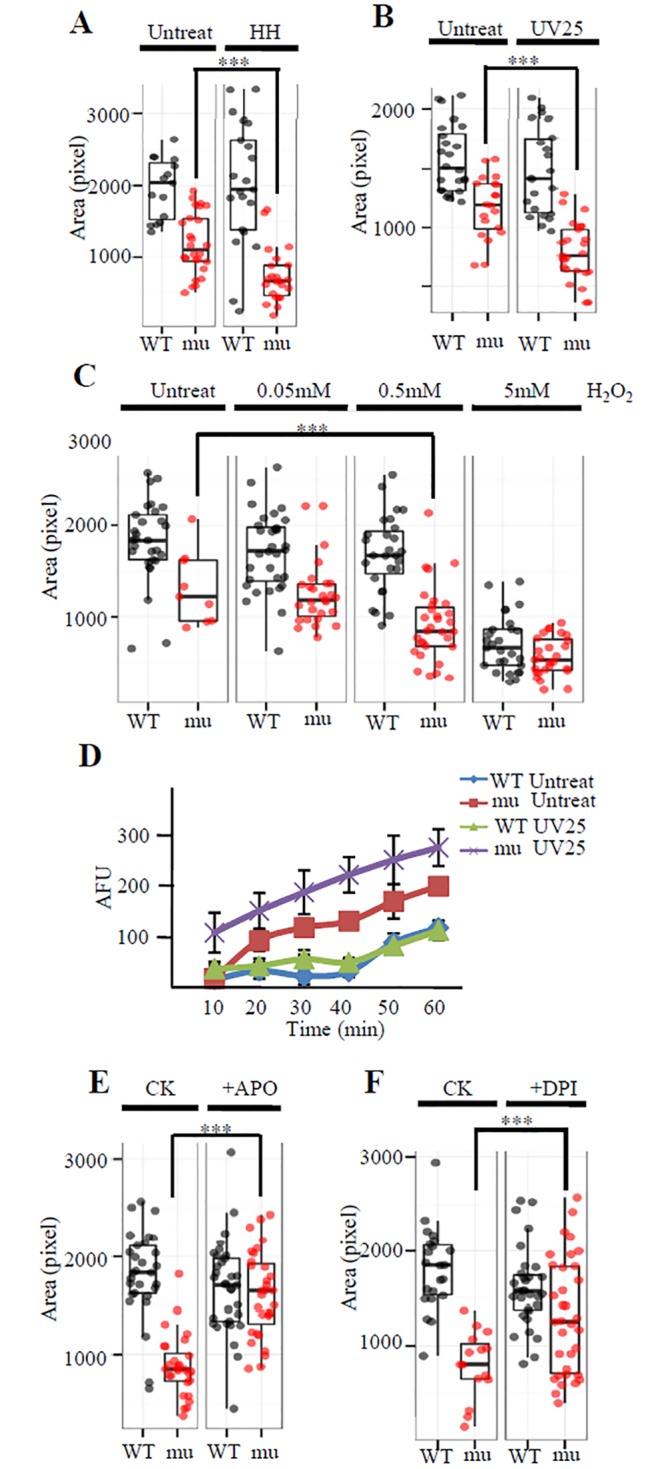
Liver development in the maternal-zygotic *leg1a*^*zju1*^ mutant is amenable to different stresses. (A and B) When growing in high temperature (32°C) and high density (200 embryos per 10-cm diameter Petri dish) (HH) starting from 10 hpf till to 3.5 dpf(A) or briefly treated with 2.5 mJ UV/cm^2^ (UV25) at 24 hpf(B) the maternal-zygotic *leg1a*^*zju1*^ embryos (mu) consistently exhibited a more severe small liver phenotype when compared with the untreated mutant embryos. (C) Comparison of liver development in WT and the maternal-zygotic *leg1a*^*zju1*^(mu) embryos treated with 0.05, 0.5 and 5 mM H_2_O_2_ at 24 hpf for half an hour. (D) Upon UV25 treatment, the maternal-zygotic *leg1a*^*zju1*^ (mu) embryos accumulated a higher level of ROS when compared to the treated WT or untreated mutant embryos within 60 min. (E and F) Incubation with APO (E) and DPI (F), two inhibitors of Duox/Nox enzyme for O_2_^-^ biosynthesis, prior to UV25 treatment prevented the effect of UV on liver development in the maternal-zygotic *leg1a*^*zju1*^(mu) embryos. Liver size was measured at 3.5dpf. Quartile boxplot was used to present the data. Each dot represents the liver size of an individual embryo. CK, the APO or DPI untreated control group. ***, p<0.001.

These results suggest that liver development in the maternal-zygotic *leg1a*^*zju1*^ mutant is amenable to its living environment. We tested this hypothesis by growing fish in different mild stress conditions. Growing maternal-zygotic *leg1a*^*zju1*^ mutants in relative high temperature (32°C) and high density (200 embryos per 10-cm diameter Petri dish) (Fig 2A) or briefly treating the maternal-zygotic *leg1a*^*zju1*^ mutants at 24 hpf with 2.5 mJ/cm^2^ ultraviolet (UV) irradiation (UV25) (Fig 2B, S2A and S2B Fig) sharply increased the proportion of the mutant embryos displaying the small liver phenotype at 3.5 dpf. High density alone also caused a small liver phenotype to the maternal-zygotic *leg1a*^*zju1*^ mutants (S2C Fig). In addition, we found that incubating the zygotic *leg1a*^*zju1*^ mutant in the egg water containing a mild but not lethal dose of H_2_O_2_ (0.5mM) also led to the small liver phenotype (Fig 2C). Interestingly, the maternal-zygotic *leg1a*^*zju1*^ mutant embryos did not exhibit a small liver phenotype at 3.5 dpf when they were grown in the egg water containing 0.5% or 1% ethanol starting at 24 dpf (S2D Fig), a concentration range not causing overall abnormality [21]. UV25 treatment also enhanced the small liver phenotype in *leg1a*^*zju2*^, another mutant allele of the *leg1a* gene (S2E Fig).

UV treatment, high temperature, andH_2_O_2_ treatment all would lead to oxidative stress [22]. To find out whether the maternal-zygotic *leg1a*^*zju1*^ mutant is compromised in scavenging ROS caused the oxidative stress, we compared the ROS level at different time points between the UV25 treated WT and maternal-zygotic *leg1a*^*zju1*^ embryos by DCFH-DA [23]. The result showed that the maternal-zygotic *leg1a*^*zju1*^ embryos accumulated a higher ROS level at all time points examined within the first hour after UV treatment(Fig 2D). We wondered whether the development of the small liver phenotype in *leg1a* mutants could be prevented by blocking the production of reactive oxygen species (ROS). Diphenyleneiodonium (DPI) and apocynin (APO) are two specific inhibitors of the Duox/Nox enzyme often used to block the production of ROS[24,25]. We treated the maternal-zygotic *leg1a*^*zju1*^ mutants with DPI or APO one hour prior to the UV25 treatment and found that both DPI and APO prevented the mutants from developing the small liver phenotype (Fig 2E and 2F).

### Leg1a protects liver development under stress conditions

**Fig 3:**
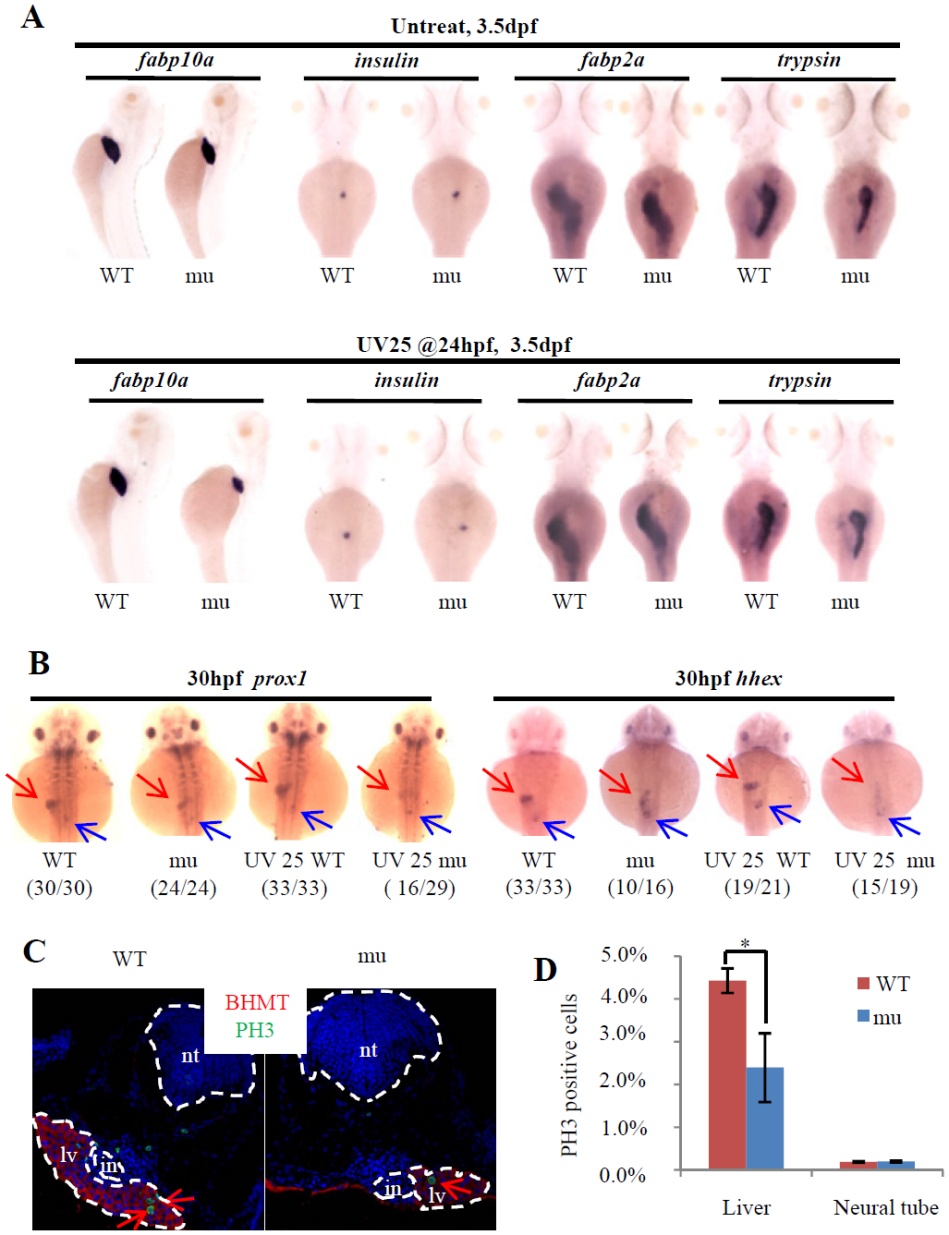
Loss-of-function of Leg1 blocks the liver bud formation. (A) Loss of maternal zygotic Leg1a affects the liver development. Embryos were treated with or without UV25 at 24hpf, WISH was performed to assess the development of the liver (*fabp10a*), exocrine (*trypsin*) and endocrine (*insulin*) pancreas and intestine (*fabp2a*) in the maternal-zygotic *leg1a*^*zju1*^(mu) embryos at 3.5 dpf. At least three independent WISH was performed, each time with 24–31 embryos for each sample, and representative embryos were shown. (B) WISH using *prox1* and *hhex* to examine liver bud formation at 30 hpf in embryos treated with or without UV25 treatment at 24 hpf. Representative pictures in each group are presented. The number of embryos exhibiting the phenotype over total embryos examined are shown in the bracket. Red arrow: liver bud, blue arrow: pancreatic bud. (C and D) Images (C) and statistical analysis (D) of PH3 immunostaining to compare cell proliferation of hepatocytes in WT and maternal-zygotic *leg1a*^*zju1*^ embryos (mu) after UV25 treatment. BHMT is an enzyme highly expressed in the liver and was used to mark out the hepatocytes. DAPI was used to stain the nuclei. Red arrows indicate PH3 positive cells in the liver. PH3 positive cells in the neural tube in WT and the mutant were also recorded in parallel. *, *p*<0.05. in, intestinal tube, lv, liver, nt, neural tube.

Liver, exocrine pancreas and intestine are all derived from the endoderm [26]. Previous genetic screening found that mutants with defects in liver development often showed defective development of the exocrine pancreas and/or intestine [27,28], likely because liver and exocrine pancreas share common progenitors [29,30]. Leg1a expression is enriched in the embryonic liver, but meanwhile, Leg1a is also a secretory protein [15]. Considering this fact, we wanted to determine whether *leg1a*^*zju1*^ also affects development of the pancreas and other digestive organs. We used *fabp10a*, *trypsin*, *insulin* and *fabp2a* probes in WISH to mark the liver, exocrine pancreas, endocrine pancreas and intestine, respectively. Interestingly, it appeared that UV25 treatment drastically reduced the liver size, but only subtly affected the exocrine pancreas development and did not show observable effect on the intestinal tube development in the maternal-zygotic *leg1a*^*zju1*^ mutant (Fig 3A). We then used *prox1* and *hhex*, two earlier hepatic markers, in WISH to examine the effect of UV25 treatment on liver bud formation at 30 hpf[13]. The result showed that UV25 treatment halted liver bud formation in most of the mutant embryos but not the WT (Fig 3B).

A TUNEL assay did not reveal any obvious differences in the apoptotic activity between the UV25-treated WT and mutant liver cells (S3 Fig, total 12cryosections from 6 embryos examined). Immunostaining of phosphorylated histone 3 (PH3, a molecular marker for cell proliferation) showed that *leg1a*^*zju1*^ liver cells (defined by immunostaining of the hepatic marker Betaine homocysteine S-methyltransferase (BHMT), in red) contained significantly fewer (*p*<0.05) PH3 positive cells (12 of 604 total cells counted or 2.4%, data obtained from 6 embryos) when compared with those in the WT (30 of 684 total cells counted or 4.43%, data obtained from 6 embryos) at 54 hpf after UV25 treatment (Fig 3C and 3D). Therefore, the maternal-zygotic *leg1a*^*zju1*^ mutant develops a small liver phenotype under UV stress due to cell cycle arrest.

### Leg1a activates the Erk pathway to promote liver development

**Fig 4:**
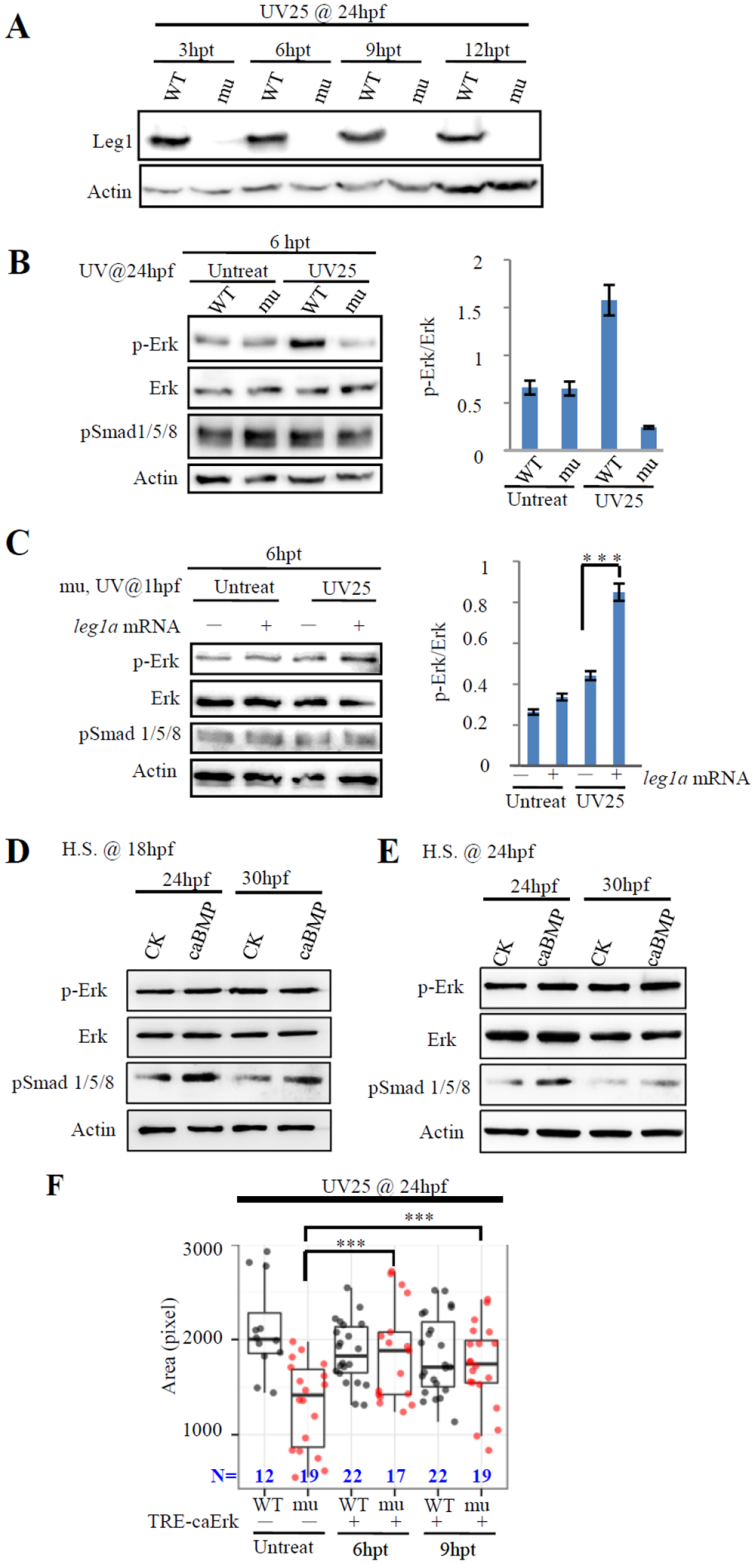
Leg1 promotes the phosphorylation of Erk. (A) UV25 treatment did not affect the Leg1 protein level. WT and maternal-zygotic *leg1a*^*zju1*^ embryos were treated with UV25 at 24 hpf. UV25 treated embryos were harvested for total protein extraction for western blot analysis of Leg1 at 3, 6, 9 and 12 hours post treatment. (B)24-hpf WT and maternal-zygotic *leg1a*^*zju1*^ mutant (mu) embryos were treated with or without UV25. Total protein was extracted at 6 hours post treatment (hpt) and subjected to western blot analysis of p-Erk, total Erk, and pSmad 1/5/8. (C) *leg1a*^*zju1*^ embryos at one-cell stage were injected with or without *leg1a* mRNA, then treated with or without UV25 at 1 hpf. Total protein was extracted at 6 hours post treatment (hpt) and subjected to western blot analysis of p-Erk, total Erk, and pSmad 1/5/8. (D and E) Over-expression of Bmp2a does not activate the phosphorylation of Erk. *Tg(hsp70l*:*bmp2b)* embryos were heat-shocked at 18 hpf (D) or 24 hpf (E). Total protein was extracted from embryos 6 or 12 hours post heatshock and subjected to western blot analysis of pSmad1/5/8, p-Erk and total Erk. Over-expression of Bmp2 by heatshock increased only the level of pSmad1/5/8 and not that of p-Erk. CK, wild type embryos; caBMP, *Tg(hsp70l*:*bmp2b)* embryos.(F) *TRE-caErk* plasmid was injected into WT and mutant (mu) embryos at one-cell stage. Injected embryos were treated with Dox at 24 hpf for 6 or 9 hours (6hpt or 9hpt) and were then harvested at 3.5 dpf for WISH using the *fabp10a* probe.(A-E) Western blot was repeated six times for (A), four times for (B), five times for (C) and twice for (D) and (E). Actin was used as a loading control. ***, *p*<0.001.

One possible explanation for the inhibitory effect of UV25 treatment on liver development in the maternal-zygotic *leg1a*^*zju1*^ mutant is that UV25 treatment induces the expression of Leg1. However, we observed that the UV- or H_2_O_2_-treatment of the WT embryos at 24 hpf did not cause significant changes to the levels of total *leg1* transcripts at 3 and 6 hours after treatment (S4 Fig). UV25 treatment of the WT embryos at 24 hpf neither affected the level of total Leg1 protein at 3, 6, 9 and 12 hours after treatment (Fig 4A). In zebrafish, 24–34 hpf is a crucial stage for hepatogenesis when signaling molecules including FGF, BMP, Wnt2bb and RA orchestrate the initiation of the liver bud [13,31–33]. Based on all of the above, we speculated that the signaling pathway promoting cell proliferation is probably impaired due to loss of the maternal-zygotic Leg1a. This prompted us to investigate whether Leg1, being a secretory protein, is involved in known signaling pathways. We treated 24hpf WT and maternal-zygotic *leg1a*^*zju1*^ mutant embryos with UV25 and found that UV25 treatment up-regulated the level of p-Erk in WT but showed an inhibitory effect on the mutant at 6 hours post treatment (i.e. at 30 hpf) (Fig 4B). Notably, UV25 treatment did not affect the Bmp signaling as indicated by the level of pSmad1/5/8 (Fig 4B). Importantly, upon UV25 treatment, Leg1a over-expression by *leg1a* mRNA injection at one-cell stage increased the level of p-Erk but did not alter the level of pSmad1/5/8 (Fig 4C). Considering that the activation of the expression of Bmp2 by heatshock of Tg(*hsp70l*:*bmp2b*) [34] embryos at 18 or 24 hpf increased only the level of pSmad1/5/8 and not that of p-Erk(Fig 4D and 4E), we speculated that Leg1 does not signal through the Bmp pathway but probably through the Erk signaling pathway to protect liver development under stress conditions.

To test whether Leg1 acts through the Erk-signaling pathway to protect liver development, we generated a constitutively active form of Erk mutant (caErk) by substituting L^84^ to P^84^ (L84P), S^162^ to D^162^ (S162D), D^330^ to N^330^ (D330N) simultaneously[35]. It has been shown that over-activating Erk signaling at the early stage (up to 80% epiboly) negatively regulates the endoderm formation[36]. Indeed, we found that injection of caErk mRNA into one-cell stage embryos caused small liver both in WT and mutant embryos (S5A Fig). To overcome the effect of Erk-signaling on early embryogenesis we injected *caErk* mRNA or *fgf8* mRNA into the yolk at 22hpf and treated the embryos with UV25 at 24hpf. The effectiveness of this way of injection is demonstrated by the fact that the injected Cy3-labled oligo-dT can successfully reach to the prospective liver bud region (S5B Fig). We found that such injection rescued the mutant liver development to a great extent (S5C Fig). Next, we cloned the *caErk* gene downstream of the doxycycline (Dox) inducible promoter tetracycline response element(TRE)promoter[37,38](S6A Fig). The expression of caErk is effectively induced by Dox after a low dosage (10 pg) of plasmid injection although a weak leakage of the TRE promoter was observed (S6B Fig). The *TRE-caErk* plasmid (10 pg) was injected into one-cell stage maternal-zygotic *leg1*^*zju1*^mutant embryos and the injected embryos were treated with UV25 at 24 hpf followed immediately by addition of the drug Dox (final concentration 30 μg/mL). The liver development in these embryos was examined with the *fabp10a* probe at 3.5 dpf. The result showed that induction of the caErk expression between 24hpf and 33hpf achieved a significant rate of rescue of the liver growth in maternal-zygotic *leg1*^*zju1*^mutant (Fig 4F) while overall features of the injected embryos appeared relatively normal (S6C Fig).

### Leg1 is modified by glycosylation at N^70^

**Fig 5:**
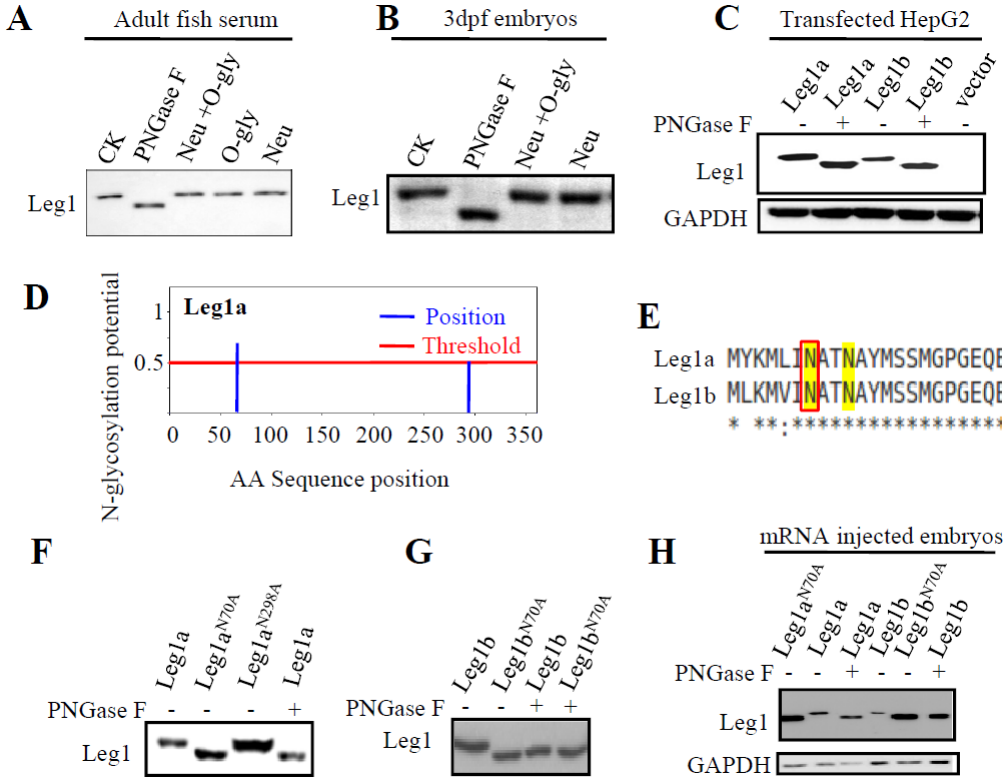
Leg1 is modified by glycosylation at N^70^. (A and B) Checking the status of glycosylation in the endogenous Leg1 by treating protein extracted from the serum (A) or 3dpf embryos (B) with PNGase F, neuraminidase (Neu), or Neu plus endo-α-N-acetylgalactosaminidase (O-gly), respectively. CK, enzyme untreated control sample.(C) Checking the status of glycosylation of Leg1a or Leg1b in plasmid transfected HepG2 cells by treating the protein samples with PNGase F.(D-H) Determination of N^70^ as the glycosylation site in Leg1. Prediction of the site(s) of glycosylation in Leg1a based on the Asn-Xaa-Ser/Thr motif using the software program NetNGly (http://www.cbs.dtu.dk/services/NetNGlyc/) (D).N^70^ is predicted to be a putative N-glycosylation site by NetNGly in both Leg1a and Leg1b (E). Mutating N^70^ to A^70^ in either Leg1a (F,H) or Leg1b (G,H) caused a gel mobility shift of Leg1 like that did the PNGase F-treated Leg1. Total protein was extracted from the plasmid transfected HepG2 cells (F and G) or mRNA injected embryos (H). Western blot was repeated twice for (A), three times for (B), (C) (F), (G) and four times for (H).

We showed previously that Leg1 is a classical secretory protein [15]. Because glycosylation is a common modification for a secretory protein [39], we checked whether Leg1 is also modified by glycosylation. There are two types of glycosylation, N-glycosylation and O-glycosylation [40]. N-glycosylation can be cleaved by PNGase F [41]whereas O-glycosylation can be cleaved by the combination of endo-α-N-acetylgalactosaminidase plus neuraminidase [42]. We previously reported that adult fish serum contains a high level of Leg1 [15]. We used these enzymes to treat the serum protein and also total protein extracted from embryos at 3dpf, respectively, and found that only PNGase F treatment caused a band shift (Fig 5A and 5B). Because both *leg1a* and *leg1b* are expressed in the adult liver to produce the serum Leg1 [15], the fact that PNGase F treatment caused a clear band shift to total Leg1 protein from the serum suggests that both Leg1a and Leg1b are modified by N-glycosylation. To confirm this hypothesis, *leg1a* and *leg1b* were cloned into the expression vector PCS2^+^, and the obtained plasmids were used to transfect the human liver cancer cell line HepG2. PNGase F treatment caused a band shift to both the expressed Leg1a and Leg1b in HepG2 (Fig 5C).

To determine which amino acid residue is glycosylated in Leg1a and Leg1b, we used a web-based platform, NetNGly (http://www.cbs.dtu.dk/services/NetNGlyc/), to predict the site of modification(s) based on the Asn-Xaa-Ser/Thr motif [43]. The prediction showed that the 70^th^ asparagine (N^70^) was a putative glycosylation site for both Leg1a and Leg1b (Fig 5D and 5E). For Leg1a, N^298^ was also predicted to be a candidate site for glycosylation (Fig 5D). We then mutated N^70^ and N^298^ in Leg1a to alanine (A) to obtain the *leg1a*^*N70A*^ and *leg1a*^*N298A*^ plasmids. We transfected the *leg1a* WT plasmid and the two *leg1a*^*N70A*^ and *leg1a*^*N298A*^ mutant plasmids into the HepG2 cell line, respectively, and performed western blot analysis of Leg1 in the extracted total protein at 24 hours post transfection. The result showed that *leg1a*^*N70A*^ produced a product with a mobility like that of Leg1a treated with PNGse F, whereas *leg1a*^*N298A*^ produced a product with a mobility like that by the *leg1a* WT plasmid (Fig 5F). We also mutated the N^70^ to A^70^ in Leg1b and found that Leg1b^N70A^ was no longer sensitive to PNGase F treatment and exhibited a mobility like that of Leg1b treated with PNGase F (Fig 5G). In addition, we injected *leg1a*^*N70A*^ and *leg1b*^*N70A*^ mRNA and their respective WT control mRNA into zebrafish embryos at the one-cell stage and extracted total protein at 9 hours post injection. Western blot analysis of the protein samples showed that both Leg1a^N70A^ and Leg1b^N70A^ exhibited a mobility like that of Leg1a or Leg1b treated with PNGase F (Fig 5H). All of these results demonstrated that N^70^ was the only N-linked glycosylation site for both Leg1a and Leg1b.

### N^70^-glycosylation is necessary for the secretion of Leg1b but not Leg1a

**Fig 6:**
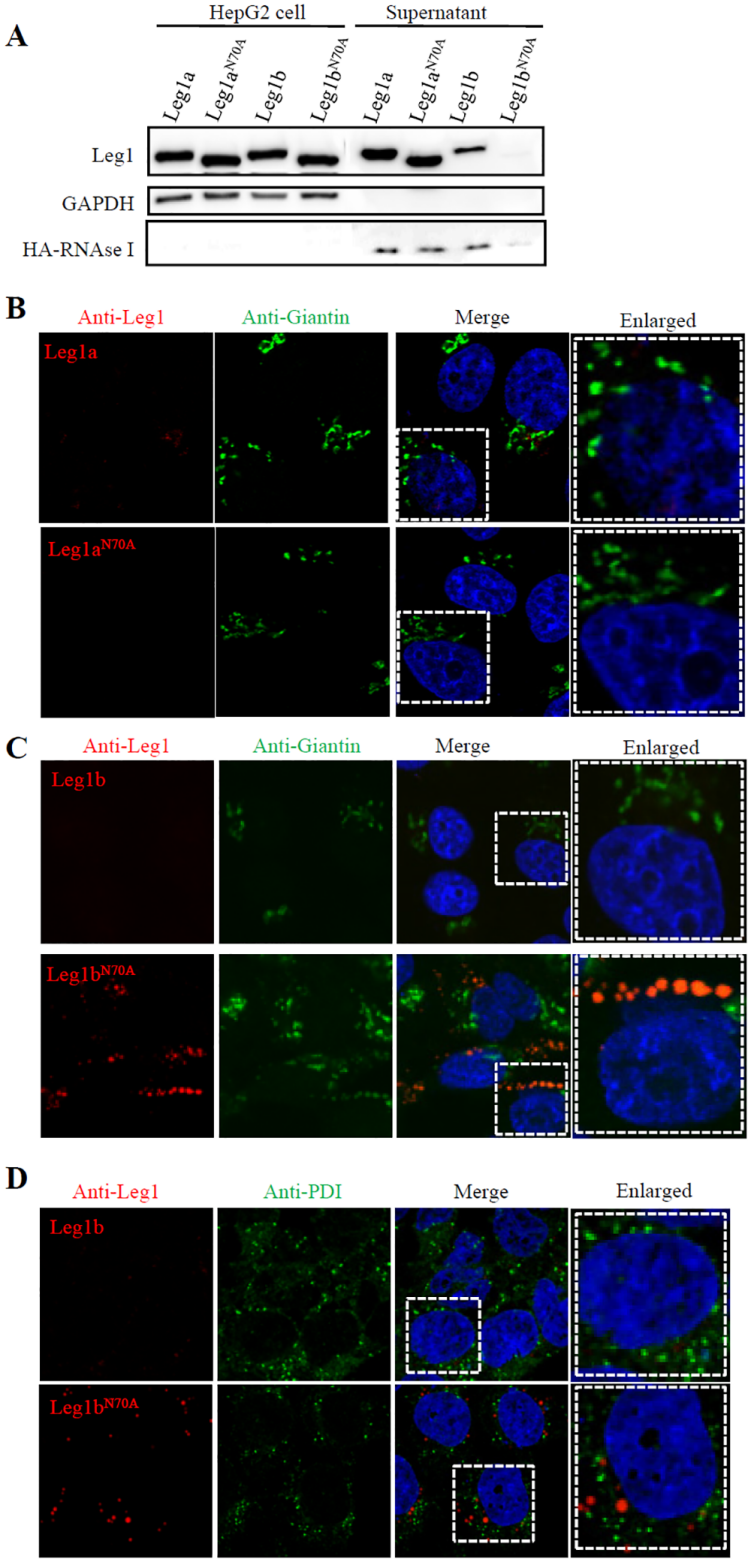
N^70^-glycosylation is necessary for the secretion of Leg1b but not Leg1a. (A) Western blot analysis of secretion of Leg1a, Leg1a^N70A^, Leg1b and Leg1b^N70A^. Mutating N^70^ to A^70^ does not affect the secretion of Leg1a^N70A^ but does affect Leg1b^N70A^. Total protein was extracted from cell pellets or supernatant. HA-tagged RNaseI was used as a control. Western blot was repeated three times.(B) Co-immunostaining of Leg1a or Leg1a^N70A^ with the *cis*- and medial-Golgi marker Giantin in HepG2 cells transfected with WT *leg1a* and *leg1a*^*N70A*^ plasmids, respectively. (C) Co-immunostaining of Leg1b or Leg1b^N70A^with Giantin showed that Leg1b is secreted (upper panels) but Leg1b^N70A^ is retained in the *cis*-Golgi network (lower panels). (D) Co-immunostaining of Leg1b or Leg1b^N70A^ with the ER marker PDI in HepG2 cells transfected with WT *leg1b* and *leg1b*^*N70A*^ plasmids, respectively. The enlarged view of the area highlighted with dashed lines are shown on the right.

Glycosylation in secretory proteins often facilitates the proper folding of the protein so that the protein can be licensed to be transported to the Golgi apparatus [44]. To assess the secretory ability of Leg1a^N70A^ and Leg1b^N70A^, WT *leg1a*, WT *leg1b*, *leg1a*^*N70A*^ or *leg1b*^*N70A*^ plasmid was each co-transfected with HA-tagged *rnasel1* plasmid into HepG2 cells. *Rnasel1* (NCBI accession no. AI476973) encodes a known secretory protein Rnasel1 [45] and was used as a control here. Total proteins were extracted from the culture medium and the cell pellet, respectively. Western blot analysis of the protein samples showed that Leg1a, Leg1b, Leg1a^N70A^ and Leg1b^N70A^ were all detected in the cell pellet fraction (Fig 6A). Leg1a, Leg1b and Leg1a^N70A^ were also detected in the culture medium fraction (Fig 6A), whereas no Leg1b^N70A^ was detected (Fig 6A). Meanwhile, we noticed that the secretion of HA-Rnase1l in the cells expressing Leg1b^N70A^ was also greatly reduced (Fig 6A).

To determine where the un-secreted Leg1b^N70A^ was located in the protein trafficking route, we co-immunostained Leg1 with ER and Golgi markers, respectively. Consistent with western blot analysis, Leg1a and Leg1a^N70A^ were secreted normally (Fig 6B), as was the WT Leg1b, which was hardly detectable in the *leg1b* plasmid-transfected cells (Fig 6C and 6D, upper panels). In contrast, Leg1b^N70A^ nicely co-localized with the *cis*-Golgi indicated by *cis*- and medial-Golgi marker Giantin(Fig 6C, lower panels) but not with the ER marker PDI [46,47](Fig 6D, lower panels). Strikingly, cells transfected with the *leg1b*^*N70A*^ plasmid appeared to harbor more *cis*-Golgi components (revealed by Giantin staining) than those in the WT *leg1b*-transfected cells (Fig 6C), indicating that the Leg1b^N70A^ mutant protein is retained in the *cis*-Golgi apparatus, which caused a traffic jam in the cells such that the secretion of Rnase1l was also severely blocked in these cells (Fig 6A). The fact that accumulation of Leg1b^N70A^mutant protein in the *cis*-Golgi but not in the ER might explain why we did not observe an activation of the markers (including Bip, Chop, and p-eIF2a) for the ER-stress response either in the cultured cells (S7A Fig) or in the maternal-zygotic *leg1a*^*zju1*^ mutants(S7B and S7C Fig).

### N^70^-glycosylation is required for Leg1a to protect liver development under stress condition

**Fig 7:**
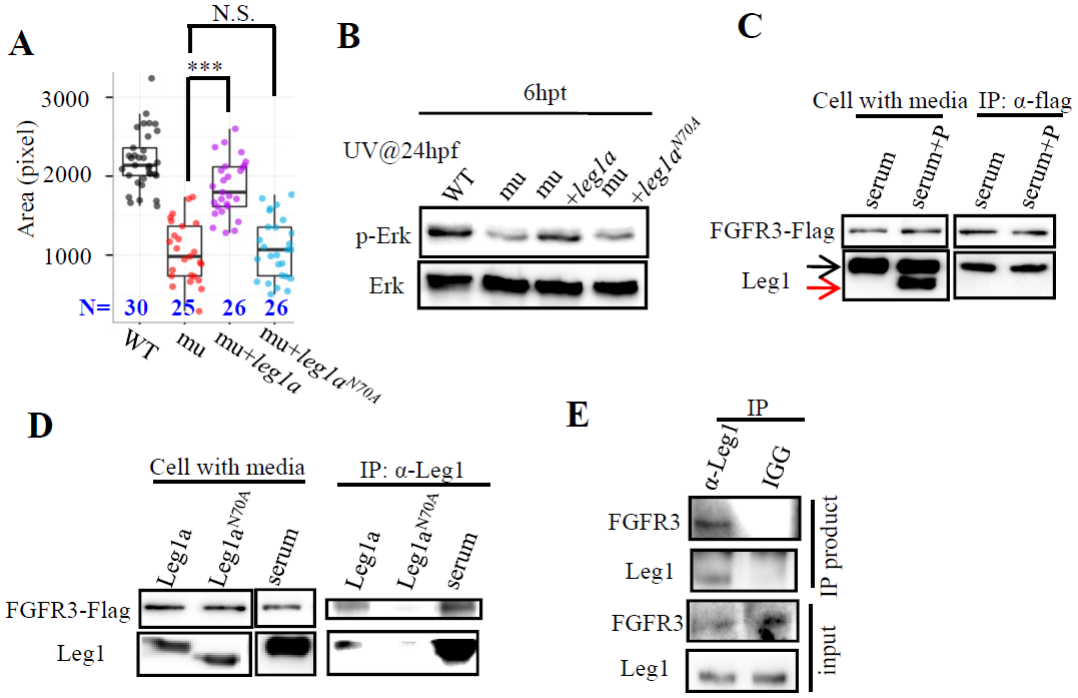
Leg1a interacts with FGFR and glycosylation at N^70^ is crucial for Leg1 to interact with FGFR3 and to protect liver development under stress conditions. (A and B) Maternal-zygotic *leg1a*^*zju1*^ embryos (mu) at one-cell stage were injected with *leg1a* or *leg1b*^*N70A*^ mRNA. Injected embryos were treated with UV25 at 24 hpf and allowed to grow to 3.5 dpf for comparison of liver development by WISH using *fabp10a* probe (A) or to 30 hpf for protein extraction for western blot analysis of p-Erk and total Erk (B). A representative data set of three independent experiments was shown. ***, *p*<0.001, N.S., not significant. (C and D) Leg1 but not de-glycosylated Leg1 interacts with FGFR3. 293T cells over-expressing FLAG-tagged FGFR3 were incubated with total serum containing Leg1 (serum) or total serum Leg1 partially de-glycosylated by PNGase F (serum+P) (C), with supernatant containing secreted Leg1a or Leg1a^N70A^ respectively from *leg1a* or *leg1a*^*N70A*^ plasmids transfected 293T cells (D). In (C), black arrow, total Leg1, red arrow, de-glycosylated Leg1. An anti-FLAG antibody was used to perform the Co-IP, FGFR3 was detected with the anti-FLAG antibody and Leg1 with the Leg1 antibody. (E) Co-IP assay of interaction between Leg1a and FGFR3 in zebrafish. 200pg *leg1* mRNA and 400 pg*fgfr3* mRNA were co-injected into one-cell stage embryos. Total protein was harvested 7 hours after injection and was subjected to Co-IP using the Leg1 antibody or mouse IGG antibody. FGFR3 was detected by an anti-FRFR3 antibody. Western blot was repeated three times for (B), four times for (C), three times for (D) and (E).

Next, we tested whether N^70^ glycosylation in Leg1a is required to promote liver development by injecting *leg1a* and*leg1a*^*N70A*^ mRNA, respectively, into the maternal-zygotic *leg1a*^*zju1*^ mutant embryos at the one-cell stage (S8 Fig). These injected embryos were briefly treated with UV25. WISH analysis using the *fabp10a* probe showed that *leg1a*^*N70A*^ mRNA injection failed to rescue the mutant liver development (Fig 7A). In fact, Leg1a^N70A^ was greatly compromised in promoting Erk phosphorylation under UV25 (Fig 7B).

### Leg1a interacts with FGFR3 that depends on Leg1a N^70^-glycosylation

FGFis a key effector of the Erk signaling pathway. We wanted to determine whether Leg1 interacts with the FGF receptor (FGFR) to activate the phosphorylation of Erk. Extracted serum protein containing Leg1a and Leg1b (total Leg1) was incubated with human 293T cells transfected with a plasmid expressing FLAG-tagged FGFR3. Co-immunoprecipitation (Co-IP) analysis showed that Leg1 interacted with FGFR3 (Fig 7C). To determine whether N^70^-glycosylation is necessary for Leg1 to bind to FGFR3, we treated total serum proteins (containing both Leg1a and Leg1b) with PNGase F to get a mixture of Leg1 and de-glycosylated Leg1 under the undenaturized condition (Fig 7C, left panels). The mixture of Leg1 plus de-glycosylated Leg1 was incubated with 293T cells expressing FGFR3. Co-IP analysis showed that only Leg1 but not the de-glycosylated Leg1 interacted with FGFR3 (Fig 7C, right panels). We also over-expressed Leg1a and Leg1a^N70A^ in 293T cells (Fig 7D, left panels) and harvested the culture medium containing Leg1a or Leg1a^N70A^ to incubate with 293T cells overexpressing FGFR3, respectively. Co-IP showed that Leg1a but not Leg1a^N70A^ interacted with FGFR3 (Fig 7D, right panels). In zebrafish, FGFR3 was co-immunoprecipitated by the Leg1 antibody when Leg1a and FGFR3 were co-expressed by their mRNA co-injection (Fig 7E).

The zebrafish transgenic line *Tg(hsp70*:*dnfgfr1-gfp)* expresses the dominant-negative Fgfr1(dn-Fgfr1) by the *hsp70*heakshock promoter[48]. The expressed dn-Fgfr1, whose tyrosine kinase domain is replaced by GFP (as a reporter of the transgenic embryos), can form heterodimer with all FGFR subtypes so that to block the FGF signaling. When this line was treated with UV25 only we found that the level of p-Erk was increased (S9 Fig). However, when the embryos were heat-shocked at 22 hpf to express dn-Fgfr1 followed by treatment with UV25 at 24 hpf, the effect of UV25 on activation of p-Erkin the GFP^+^ embryos was down-regulated to a similar level to that observed in the UV25 untreated GFP^+^ embryos. This result further suggests that the activation of Erk by UV25-treatment is through the FGF pathway,

### Compensatory mechanism is activated in *leg1a*^*zju1*^ mutant

**Fig 8:**
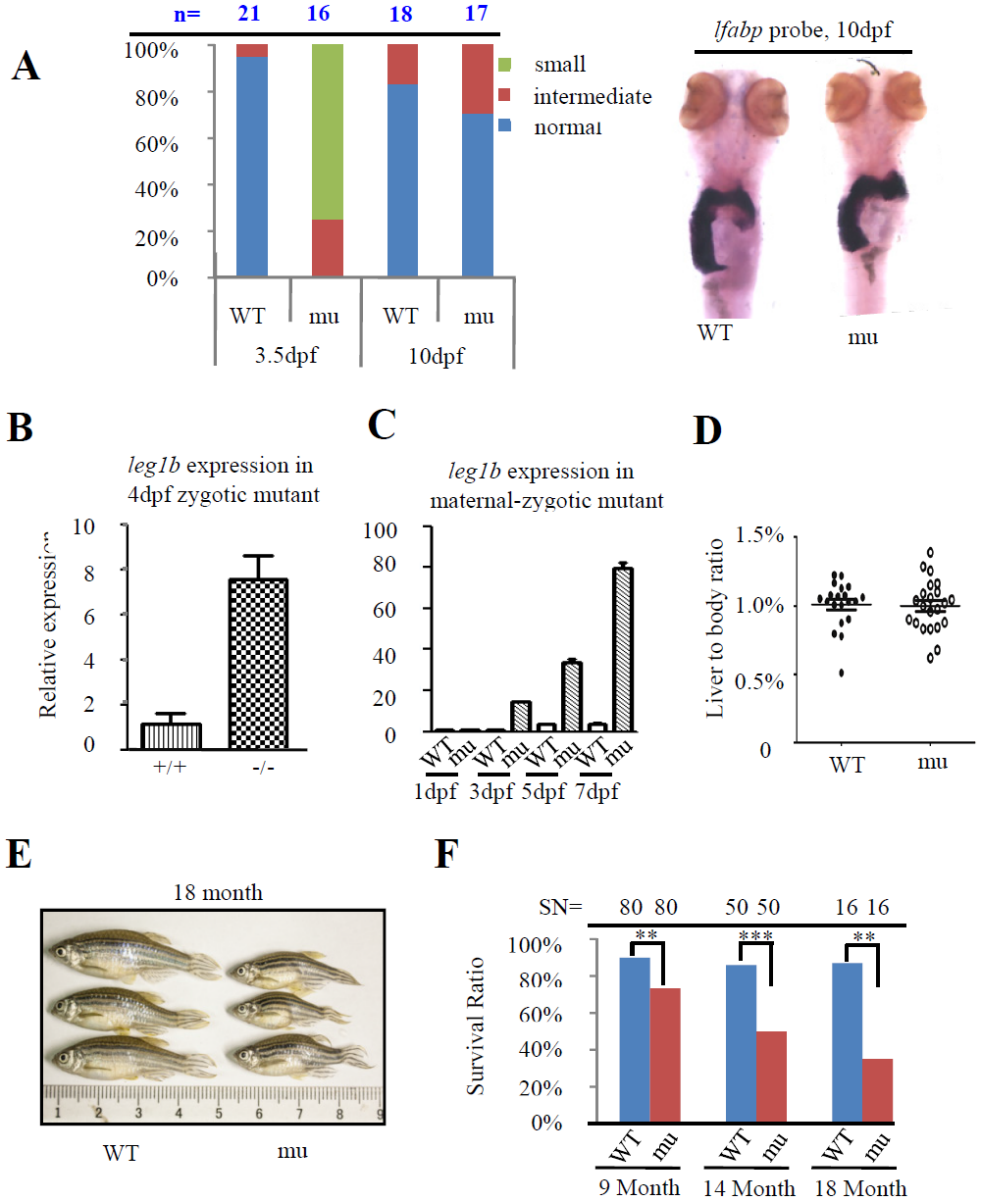
The compensatory mechanism is activated in *leg1a* mutant. (A) WISH analysis of the liver sizes in WT and maternal-zygotic *leg1a*^*zju1*^(mu) at 3.5 and 10 dpf using the *fabp10a* probe. Left panel: histogram showing the percentage of normal, intermediate and small liver in the WT and *leg1a* mutant embryos at 3.5 and 10 dpf. The number of embryos (n) was shown on the top. Right panel: representative image of 10-dpf embryos after WISH. (B and C) *leg1b* expression in the zygotic mutant embryos at 4 dpf (B) and maternal-zygotic mutant embryos at 1, 3, 5 and 7dpf(C). (D) Comparison of the liver to body ratio between WT and *leg1a*^*zju1*^mutant in the adult fish. Each dot represents the ratio for an individual adult fish. Error bar stands for the standard error. (E) Representative images of the WT and *leg1a*^*zju1*^mutant adult fish. (F) Survival rate of the WT and *leg1a*^*zju1*^mutant adult fish aged at 9, 14 and 18 months. The starting number of the larvae fish for each counting (SN) are shown on the top.

Considering the importance of the liver for a living organism and the viable and fertile nature of the*leg1a* mutant, we wondered whether the small liver in the *leg1a* mutant would be recovered to normal during later growing stages. We treated WT and maternal-zygotic *leg1a*^*zju1*^ mutant with UV25 at 24hpf, and check the liver size at 3.5dpf and 10dpf. While, as expected, almost all the maternal-zygotic *leg1a*^*zju1*^ mutant displayed a small liver compared to the WT at 3.5dpf, the liver sizes in the mutants were recovered to normal at 10dpf (Fig 8A). However, the maternal-zygotic *leg1a*^*zju1*^ mutant exhibited a lower survival rate ~32% (28/87) when compared to 66% (50/76) for the WT counted at 10 dpf. Examining the expression of *leg1b*, the homolog of *leg1a*, in the *leg1a*^*zju1*^ mutant revealed that the levels of *leg1b* transcripts were up-regulated both in zygotic homozygous mutant at 4 dpf(Fig 8B) and maternal-zygotic mutant at 3-, 5-, and 7-dpf (Fig 8C). WISH using the *leg1* probe also showed that the total *leg1* transcripts in the maternal-zygotic *leg1a*^*zju1*^ mutant was enriched in the liver (S10B Fig).These data suggest that the compensatory mechanism[49] is activated in the *leg1a*^*zju1*^ mutant to support the liver development at the later stages. At the adult stage, although the liver to body ratio of the *leg1a*^*zju1*^ mutant fish did not show significant difference to that of the WT fish (Fig 8D) the *leg1a*^*zju1*^ mutant fish exhibited a shorter stature and higher mortality compared to the WT fish (Fig 8E and 8F), suggesting that the Leg1a anti-stress pathway also functions in the adult fish and that theLeg1bonly partially compensates for the function of Leg1a.

## Discussion

In addition to the precise spatial and temporal control of genetic programs instructing oganogenesis, successful completion of organogenesis also relies on the maintenance of an optimal environment through the elimination or neutralization of the stress-induced harmful reagents, and how this is achieved is of tremendous interest in the field of developmental biology [50]. Although undergoing external embryogenesis, teleost fish harbor a robust genetic program dictating liver development as long as any environmental change, including temperature or natural UV irradiation, is not detrimental. It is therefore of interest to explore the mechanism(s) behind this phenomenon. We showed that Leg1 plays a unique role in protecting liver development under different stress conditions by serving as a secretory signaling molecule/modulator to activate the Erk pathway. This finding may explain the adaption of teleost fish in coping with environmental changes.

The process of liver organogenesis is governed by key transcription factors (e.g., HNF, GATA, Prox and Hhex) and signaling molecules (e.g. FGF, Bmp and Wnt) [1–3,10]. Meanwhile, each of these stages has to deal with the oxidative stress constantly imposed intrinsically or externally. In zebrafish, hepatoblasts are specified at around 24 hpf and start to form the liver primordium at around 30 hpf. Both FGF and Bmp play crucial roles during this period [10,31,32]. In general, FGF acts through the FGFR-RAS-ERK signaling pathway [51], Bmp through activation of Smad1/5/8 phosphorylation [52] and Wnt2bb through the β-catenin-TCF pathway to control organ/tissue development, respectively [53]. It is envisaged that molecules mediating anti-oxidative stress during liver organogenesis might act as a tuner of the pathways controlling cell proliferation or elimination. Based on the facts that 1) Leg1a expression is enriched in the yolk syncytial layer between 24–48 hpf(S10A Fig)and this layer is directly exposed to external stress such as UV irradiation or low level of oxygen, 2) Leg1a expression is obviously enriched in the embryonic liver at 48 hpf, 3) Leg1a is a secretory protein, 4) the *leg1a*^*zju1*^ maternal-zygotic mutant exhibited a small liver only under the cold season, UV irradiation, high temperature, or H_2_O_2_ treatment, and 5) the small liver phenotype was rescued by the antioxidant chemicals DPI and APO, we conclude that Leg1a defines a novel anti-stress pathway to protect the liver development. Besides, we noticed that the *leg1a*^*zju1*^ maternal-zygotic adults displayed a shortened body length and reduced survival rate, suggesting that the Leg1-meidated anti-stress pathway is also necessary for wellbeing of an adult fish.

Then, the question is how there is a season in a fish facility which is maintained at relatively constant temperature throughout the year? Since the small liver exhibited by the maternal-zygotic *leg1a*^*zju1*^ mutant is ROS-dependent we speculate that the difference in the oxygen content in the fish water in different seasons might be the cause of the stress-related phenotype although the temperature is maintained in the facility. We know that the oxygen content in the water is related to atmospheric pressure and that the atmospheric pressure is higher in the cold seasons and lower in the warm seasons. We checked the weather record between Dec 30, 2013 to Jan 30, 2015 in Hangzhou and plotted the liver size against the record of atmospheric pressure. The small liver phenotype in *leg1a* mutant nicely correlates with high atmospheric pressure in the cold seasons (S1D Fig). However, we cannot exclude other possibilities at this moment.

Utilizing specific morpholinos (MOs), we previously showed that *leg1a* is required for liver bud outgrowth[15]. However, zygotic *leg1a*^*zju1*^ mutants do not show this phenotype, and maternal-zygotic *leg1a*^*zju1*^ mutant phenotype is milder than the phenotype generated by the leg1a-MO. The discrepancy between the phenotype caused by MO-injection and the phenotype exhibited by a loss-of-function mutant is indeed a concern in the zebrafish community. The explanations for the discrepancy observed could be: 1) previous used leg1-MO might have yielded an off-target effect on the liver development in the morphants, this fits with the observation that *leg1a* or *leg1b* mRNA or even combination of *leg1a* and *leg1b* mRNA only partially rescued the morphant small liver phenotype[15]; 2) injecting morpholino itself works as a stress cue to induce the small liver phenotype when Leg1 is knocked down; and 3) since the *leg1b* gene is still intact in the *leg1a*^*zju1*^ mutant, the mild phenotype exhibited by the maternal-zygotic *leg1a*^*zju1*^ mutant might be due to the functional compensation by Leg1b[49]. To narrow down the possibilities, we tried to get the *leg1a* and *leg1b* double knockout mutant, however, failed in obtaining such double mutant. We also injected standard control morpholino (ST-MO, derived from human *β-globin* antisense morpholino) into the *leg1a*^*zju1*^ mutant embryos and found that ST-MO did not enhance the *leg1a*^*zju1*^ mutant phenotype (S11 Fig). We then compared the expression of *leg1b* in the WT and *leg1a*^*zju1*^ and found that the expression of *leg1b* is elevated in the *leg1a*^*zju1*^ mutant embryos. These data suggest that the expression of *leg1b* is mobilized to compensate, at least partially, for the loss of function of Leg1a in the *leg1a*^*zju1*^ mutant.

Mechanistically, it appears that Leg1a does not signal through the Bmp pathway because Leg1a over-expression does not promote the phosphorylation of Smad1/5/8 as done by the over-expression of Bmp at 18 or 24 hpf. However, Leg1a over-expression does promote the phosphorylation of Erk upon UV25 treatment. Furthermore, UV-treatment caused up-regulation of the phosphorylation of Erk in WT but not in the maternal-zygotic *leg1a*^*zju1*^ mutant. In addition, as revealed by immunostaining, it appeared that more p-Erk cells were in the WT endoderm than that in the maternal-zygotic *leg1a*^*zju1*^ mutant after UV25 treatment (S12 Fig). The intriguing question is why Leg1a promotes Erk phosphorylation after UV exposure. We speculate that it maybe because UV causes certain modification or conformation change to Leg1a that facilitates the interaction between Leg1a and Fgfr3 to promote Erk phosphorylation. Nevertheless, these data suggest that Leg1a signals through the Erk pathway. Since FGF is a key effector of the Erk pathway and is essential for liver development, our data suggest that there might be a crosstalk between the FGF and Leg1-meidated anti-stress signaling pathways. Therefore, it is of great interest to determine how Leg1 promotes Erk phosphorylation in the future. For example, being a secretory protein, does Leg1a have its own receptor or shares the FGF receptor to mediate its activity? If Leg1a does share the FGF receptor with FGF, then which type of receptor do they share? Or does Leg1a simply serve as an agonist to facilitate the binding of FGF to its receptor? Leg1 is an evolutionally conserved protein across the vertebrates[15]. A recent report showed that Leg1 homologs in monotreme is highly expressed in monotreme milk and appears to be modified by N-glycosylation[17]. This implies that the tissue expression specificity and function of Leg1 might vary among different animal species. Here we showed that zebrafish Leg1a is glycosylated at N^70^. Although this glycosylation modification is not essential for the secretion of Leg1a, it is important for Leg1a in the promotion of liver development, for the phosphorylation of Erk and interaction with FGFR3. All available data have suggest that Leg1a is a novel signaling molecule/modulator, which has urged us to identify more downstream signaling molecules involved in this pathway, which may ultimately reveal the importance of this pathway in the evolution of vertebrates.

## Materials and Methods

### Ethics statement

All animal procedures were performed in full accordance to the requirement by ‘Regulation for the Use of Experimental Animals in Zhejiang Province’. This work is specifically approved by the Animal Ethics Committee in the School of Medicine, Zhejiang University (ETHICS CODE Permit NO. ZJU2011-1-11-009Y, issued by the Animal Ethics Committee in the School of Medicine, Zhejiang University).

### Fish lines and maintenance

The zebrafish (*Danio rerio*) AB strain was used as WT in this study. To generate the *leg1a* mutant, we constructed a TALEN vector against the first exon of the *leg1a* gene (Fig 1A) according to the “Unit Assembly” protocol[19]. The TALEN mRNA was synthesized using the SP6 mMESSAGEmMACHINE Kit (Ambion) and was injected into the WT embryos at one-cell stage. These embryos were bred to the adulthood as founders to mate with a WT fish. Eight embryos from each cross were genotyped using the primer pair *leg1a* 4244 Fw (CTTACAAGTTACAGCAGCTCC) and *legg1a* 7748 Rv (CACAACGGACCAGTACATCG) followed by the second primer pair TALEN ID fw (CTCCCAGAGGATGACCATGT) and TALEN ID Rv (ACTCCAGAGCGGATTCTCCT) to identify *leg1a* mutants, and the rest embryos were bred to adulthood for identification of individuals carrying the mutation. The *Tg*(*hsp70*:*dnfgfr1-gfp*) and *Tg(hsp70l*:*bmp2b)* fish lines were obtained from Dr Feng Liu. Fish was raised and maintained in the fish facility (Ai-Sheng Zebrafish Facility Manufacturer Company, Beijing, China) in Zhejiang University according to the standard procedure.

### Cell lines and plasmid transfection

HepG2 cells were grown in the DMEM medium (high glucose, GIBCO), supplemented with 10% newborn calf serum (NBCS, GIBCO). Plasmids were transfected into cells mediated with lipofectamine 2000 (InVitrogen) according to the manufacturer’s instruction. Total protein was extracted 24 hours after transfection and was subjected to western blotting analysis.

### Plasmid construction and mRNA *in vitro* transcription

The ORF region of *leg1*and *erk* was cloned into PCS2+ vector. All *leg1*and *erk* point mutations were generated by site-directed mutagenesis. The primers for *leg1* mutant used in the PCR reaction were designed by the webtoolPrimerX (http://bioinformatics.org/primerx/index.htm). The sequences of primers are listed in S1 Table. All primers used for *erk* point mutation was designed as previously described[35]. mRNAs were obtained via *in vitro* transcription using the mMessagemMachine (Ambion) according to the manufacturer’s instruction.

### Immunofluorescence staining

Cells were seeded in glass slide when the optimal cell density was achieved and fixed by 3% PFA for half an hour at 4°C. The cells were washed twice with 50mM NH_4_Cl and three times with PBS and were then penetrated with PBST (PBS+0.1% Triton X 100) for 15min and blocked with blocking buffer (5% goat serum, 5% fetal bovine serum and 2% bovine serum albumin) for 30min, sequentially. Cells were finally incubated with corresponding primary antibody and then Alexa Fluor conjugated second antibody. The samples were visualized under a confocal microscope. Leg1 antibody [54] and BHMT antibody [55] was generated as described. PDI antibody (Sigma,P7496), Giantin antibody (Abcam, ab24586), PH3 antibody (Santa Cruz, SC-8656-R), Actin antibody (Huabio, R1207-1), GAPDH antibody (Huabio, M1211-1), p-Erk antibody (Cell Signalling Technology, 9101), total Erk antibody (Cell Signalling Technology, 4695), pSmad1/5/8 (Cell Signalling Technology, 9511) antibody were purchased from the companies as indicated.

### Whole-mount *in situ* hybridization (WISH) and liver size measurement

WISH was performed as previously described [27]. *prox1*, *hhex*, *fabp10a* (*liver fatty acid binding protein 10*), *insulin* and *trypsin*, *fabp2* (*intestinal fatty acid binding protein 2*) were cloned into expression vectors, respectively [27,31]. Corresponding probes were synthesized via *in vitro* transcription and were labeled with digoxigenin (DIG, Roche, Diagnostics). Liver size was measured as previously described [54]. Briefly, liver was marked out after WISH suing the *fabp10a* probe, and imaged by Nikon AZ100 from left lateral view after aligning two eyes of the embryo vertically. The *fabp10a* signal area in each image was calculated by Nikon image system (NIS-elements D v3.0)and used as the index of the liver size.

### Glycan cleavage

PNGase F (NEB, P0704) was used to cleave N-linked glycosylation, and a combination of Endo-alpha-α-Acetylgalactosaminidase (NEB, P0733) and neuraminidase (NEB, P0720) was used to cleave *O*-linked glycosylation. All the enzyme treatment was performed according to the manufacturer’s instruction.

### Western blot

For either fish embryo or cultured cells, total protein was extracted using an extraction buffer (63mM Tris-HCl, PH6.8, 10% glycerol, 5% β-Mercaptoethanol, 3.5% SDS) containing 1X Complete (Roche, 11873580001). Western blotting was performed as described previously [27] using corresponding antibodies as indicated in the figures. Actin antibody (Huabio, R1207-1), GAPDH antibody (Huabio, M1211-1), p-Erk antibody (Cell Signalling Technology, 9101), total Erk antibody (Cell Signalling Technology, 4695), pSmad1/5/8 antibody (Cell Signalling Technology, 9511), Bip antibody (Sigma, G9043), Chop antibody (Sigma, G6916), phosphorylated eIF2a (p-eIF2a) antibody (Cell Signaling Technology, 9721S), and total eIF2a antibody (Cell Signaling Technology, 9720S), and Flag antibody (Sigma, F1804) were purchased from the companies as indicated. Signal intensity of a desired band was calculated by ImageJ software (v.1.48).

### UV and drug treatment

Embryos were treated with different dosage of UV energy supplied by Ultraviolet Crosslinker (UVP, CL-1000) at 24 hpf and then allowed to grow in the egg water. For H_2_O_2_ treatment, 24 hpf old embryos were treated with different concentration of H_2_O_2_ for half an hour. For APO and DPI treatment, embryos were incubated with 0.5 μM APO (Sigma,W508454) or 10μM DPI (Sigma, D2926) for 1 hour at 23 hpf, followed by UV25 treatment, and then allowed to grow in normal egg water. Embryos injected with TRE-caErk plasmid were treated with Dox at 24hpf, and replaced with fresh egg water at 30hpf (6hpt) and 33hpf (9hpf), respectively.

### Measurement of ROS level

ROS content measurement was performed as described previously [56] with some modifications. Briefly, embryos were sunk in 100μl of 10μM DCFH-DA(Beyotime, S0033) solution for one hour prior to UV25 treatment. For each sample, embryos were divided into four groups (containing three embryos in each group) and placed in a 96-well plate. After UV25 treatment the fluorescence signal was measured at a 10 min interval for one hour on a Synergy H1 Reader (Biotek) (excitation 485 nm, emission 560 nm).

### Co-immunoprecipitation (Co-IP)

For studying the interaction between Leg1 and FGFR3 in the cell culture system, Leg1 sample was prepared either by diluting 30μl of the fish serum with 1000μl of serum-free DMEM media (Gbico) or by transfecting 293T cells with the *leg1* plasmid (cloned into the PCS2^+^ expression vector) and collecting the culture media 30 hrs after transfection. The Leg1 samples were incubated with the 293T cells expressing FGFR3 (cells transfected with the *FGFR3* plasmid in the PLX304 expression vector, the vector is provided by Dr Bing Zhao) at 4°C for one hour. After incubation, the cells were washed with PBS for three times, and lysed with NP40 lysis buffer (50mM Tris-Hcl, PH 8.0, 150mM NaCl, 1% NP40, 2mM EDTA). For studying the interaction between Leg1 and FGFR3 in the embryos, embryos injected with *leg1a* and *fgfr3* mRNA at one cell stage were harvested at 7hpf and lysed with NP40 lysis buffer. All lysates were incubated with Leg1 antibody or Flag antibody at 4°C overnight, followed by incubation with Protein A/G argrose beads (beyotime, Cat.No.P2012) for further 2 hrs. The beads were washed with cold PBS for three times and eluted by 100mM PH2.2 glycine. The elution was subjected to western blot analysis.

### Quantitative Real Time-PCR (qPCR)

More than 50 embryos were pooled for total RNA extraction. Reverse transcription was performed by SuperScript II Reverse Transcriptase (Invitrogen, 18064–014) according to the manufacturer’s protocol. The transcribed cDNA was used as the template in qPCR with SYBR Green Master Mix (Vazyme). The CFX96 real time system (Bio-Rad) was used to obtain the threshold cycle (C_t_) value, and the relative expression of each gene was determined after being normalized to the *actin* gene. Primer pairs used are listed in S2 Table.

### Statistics

In considering of relative small sizes of samples with skewed phenotype distribution among individuals in this study, the conventional statistical analysis by showing mean and standard error/derivation is apparently not suitable for presenting the liver size measurement data. Instead, quartiles are more intuitive in presenting data with relative small sample size with skewed distributions [57]. Therefore, we used the quartile boxplot to present our data [58]. The box plot was drawn by ggplot2 [59]. Survival ratio statistical analyses were carried by Chi-squared test. Other statistical analyses were performed with the Student’s T-test. *, *p*<0.05, **, *p*<0.01, ***, *p*<0.001, N.S, no significant difference.

## Supporting Information

S1 Table Primers used to generate *leg1* mutant. (DOCX)

S2 Table Primers for qPCR. (DOCX)

S1 Fig Liver development in the maternal-zygotic *leg1a*^*zju1*^ mutant is amenable to the environmental changes. (A) Representative images of embryos after WISH using the *fabp10a* probe corresponding to the result shown in Fig 1F. (B and C) Among the 32 cases shown in Fig 1G, the number of embryos exhibiting a small versus normal liver in 5 cases recorded in cold seasons (B) and 6 cases recorded in warm/hot seasons (C) were shown. (D) Plotting the liver sizes (majority normal or majority small) against daily atmospheric pressure in Hangzhou recorded during 30/12/2013 and 20/02/2015 (obtained from http://www.wunderground.com/).
(TIFF)

S2 Fig Liver development in the maternal-zygotic *leg1a*^*zju1*^ mutant is amenable to oxidative stress. (A) Images showing an example of determining the liver size by WISH using the *fabp10a* probe. WT: wild type; mu: *leg1a*^*zju1*^ mutant; mu+UV25: *leg1a*^*zju1*^ mutant treated with UV25. (B) WT and maternal-zygotic *leg1a*^*zju1*^(mu) embryos were treated with 1 mJ/cm^2^ (UV10), 2.5 mJ/cm^2^(UV25) and 5 mJ/cm^2^ (UV50) UV at 24 hpf and grew to 3.5 dpf for WISH analysis of liver development. (C) Comparison of liver sizes between the WT and maternal-zygotic *leg1a*^*zju1*^ (mu) embryos growing in a high density condition (200 embryos per 10-cm diameter Petri dish). (D) Growing the maternal-zygotic *leg1a*^*zju1*^ (mu) embryos in the egg water containing 0.5% or 1% ethanol did not cause a small liver phenotype. (E) Upon UV25 treatment the maternal-zygotic *leg1a*^*zju2*^ embryos also exhibited a small liver phenotype at 3.5 dpf. *, *p*<0.05, **, *p*<0.01, ***, *p*<0.001, N.S., no significance.
(TIFF)

S3 Fig *leg1a*^*zju1*^ mutant does not suffer from elevated apoptosis upon UV25 treatment. Images of TUNEL assay in the 54-hpf WT and maternal-zygotic *leg1a*^*zju1*^ embryos (mu) after UV25 treatment at 24 hpf. No abnormal apoptotic activity was observed near the endodermal region including liver (lv) and intestine (in) in the maternal-zygotic *leg1a*^*zju1*^ embryos (mu) compared to the WT. 12 sections from six embryos for each genotype were examined. nc, notochord, nt, neural tube.
(TIFF)

S4 Fig The expression of total *leg1* and *leg1b* is not affected by UV25 or H_2_O_2_ treatment. The 24-hpf WT embryos were treated with UV25 or 0.5 mM H2O2. Total *leg1* or *leg1b* RNA level were measured by quantitative PCR (qPCR) using *leg1a* and *leg1b* common primers or *leg1b* specific primers, respectively. Error bar stands for the standard error. N.S., no significance.
(TIFF)

S5 Fig Leg1a protects liver development under stress conditions through Erk signaling. (A) Injection of constitutively active form of Erk (*caErk*) mRNA into one-cell stage embryos impaired liver development both in WT and *leg1a*^*zju1*^ mutant when examined with the *fabp10a* probe at 3.5 dpf. (B) 200 pg Cy3 labeled oligo-dT(50) was injected into the yolk at 22 hpf, and the Cy3 signal was checked at 27 hpf. CK, oligo-dT(50) uninjected control. (C) Embryos were injected with *caErk* or *fgf8* mRNA into the yolk at 22 hpf and were then treated with UV25 at 24 hpf. The liver development in the treated embryos at 3.5 dpf was examined with the *fabp10a* probe at 3.5 dpf.
(TIFF)

S6 Fig Construction of the *Tre-caErk* plasmid. (A) Schematic drawing showing the structure of the *TRE-caErk* plasmid. *RTTA* expression was driven by the *β-actin* gene promoter. Dox binds to RTTA and the Dox-RTTA complex binds to the TRE promoter to drive the expression of *caErk*. (B) 10 pg*Tre-caErk* plasmid DNA was injected into one-cell stage maternal-zygotic *leg1a*^*zju1*^ embryos (mu). These embryos were treated with Dox at 6 hpf and total protein was harvested at 12 hpf. The protein samples were subjected to western blot analysis. The total Erk versus Tubulin ratios were shown on the right. Dox, doxycycline. Error bar stands for the standard error. ***, p<0.001. Western blot was repeated three times. (C) Images of representative 3.5-dpf embryos after WISH using the *fabp10a* probe. Embryos was first injected with *Tre-caErk* plasmid at one-cell stage, then treated with UV25 at 24 hpf and followed by Dox treatment for 6 hours (6 hpt) or 9 hours (9 hpt). After Dox treatment, embryos were transferred to the normal egg water to grow to 3.5 dpf for WISH (n = 20).
(TIFF)

S7 Fig Leg1b hypoglycosylation and Leg1a mutation do not cause ER stress response. (A) HepG2 cells were transfected with the *leg1b*, *leg1b*^*N70A*^, and the vector plasmid DNA. Total protein was extracted 30 hours post transfection and subjected to western analysis of Bip, Chop, phosphorylated eIF2α (p-eIF2α), and total eIF2α. These ER-stress response markers were not activated by the hypoglycosylated Leg1b^N70A^. Vector, the PCS2^+^ vector transfected cell. (B) Western blot analysis of Bip, Chop, phosphorylated eIF2α (p-eIF2α), and total eIF2α in the WT and maternal-zygotic *leg1a*^*zju1*^ mutant embryos at 2 dpf and 3 dpf. (C) qPCR analysis of the transcript levels of ER-stress response markers including *atf6*, *bip*, *perk*, *chop*, *ire1a*, and *grp94* in 3 dpf WT and maternal-zygotic *leg1a*^*zju1*^ mutant embryos. Error bar stands for the standard error. Primers for analyzing these ER stress marker was as previously reported (S2 Table). Western blot was repeated three times each for A and B.
(TIFF)

S8 Fig N^70^ Glycosylation is required for Leg1a to protect liver development. Corresponding to Fig 7A and 7B. Western blot analysis of Leg1a or Leg1a^N70A^ protein in 3 dpf old maternal-zygotic *leg1a*^*zju1*^ mutant embryos injected with *leg1a* (mu+1a) or*leg1a*^*N70A*^(mu+1a^N70A^) mRNA at the one-cell stage. Protein samples from the WT and maternal-zygotic *leg1a*^*zju1*^ mutant (mu) embryos were used as controls. Western blot was repeated three times.
(TIFF)

S9 Fig Blocking the FGFR activity attenuates the activation of Erk by UV25 treatment. *Tg(hsp70*:*dnfgfr1-gfp)* embryos were heatshocked at 22 hpf to induce the expression of dominant negative FGFR1 (dn-Fgfr1). GFP signal was used to distinguish the dn-Fgfr-expressed (GFP+) and non-dn-Fgfr-expressed (GFP-) embryos. Embryos were treated with UV25 at 24 hpf, and total protein was extracted from embryos at 30 hpf (6 h post treatment) and was subjected to western analysis of the level of p-Erk. Tublin was used as a loading control. H.S., heatshock. *, p<0.05, **, p<0.01, N.S., no significance. Western blot was repeated two times.
(TIFF)

S10 Fig WISH analysis of total *leg1* expression in the WT embryo at 27 hpf (A, arrow points to the endoderm region giving rise to the liver primordium) and in the WT and maternal-zygotic *leg1a*^*zju1*^ mutant embryos at 7 dpf (B, arrow points to the liver). n = 25.
(TIFF)

S11 Fig Morpholino injection does not cause a small liver phenotype to the maternal-zygotic *leg1a*^*zju1*^ mutant. One nanolitre of 0.5 mM standard control mopholino (ST-MO) was injected into one-cell stage embryos. The liver development was examined at 3.5 dpf using the *fabp10a* probe. N.S., no significance.
(TIFF)

S12 Fig Immunostaining of p-Erk in the WT and maternal-zygotic *leg1a*^*zju1*^ mutant embryos at 27 hpf. Serial cryosections (S1 to S4) from three WT embryos (WT-1, WT-2 and WT-3) and three mutant embryos (mu-1, mu-2 and mu-3) treated with UV25 at 24 hpf were shown. DAPI was used to stain the nuclei.
(TIFF)

## References

[1] Stainier DY (2002) A glimpse into the molecular entrails of endoderm formation. Genes Dev 16: 893–907. 11959838 10.1101/gad.974902

[2] Zaret KS (2002) Regulatory phases of early liver development: paradigms of organogenesis. NatRevGenet 3: 499–512. 10.1038/nrg837 12094228

[3] Duncan SA (2003) Mechanisms controlling early development of the liver. Mechanisms of Development 120: 19–33. 12490293 10.1016/s0925-4773(02)00328-3

[4] Tao T, Peng J (2009) Liver development in zebrafish (Danio rerio). JGenetGenomics 36: 325–334. 10.1016/S1673-8527(08)60121-6 19539242

[5] Gordillo M, Evans T, Gouon-Evans V (2015) Orchestrating liver development. Development 142: 2094–2108. 10.1242/dev.114215 26081571 PMC4483763

[6] Lee CS, Friedman JR, Fulmer JT, Kaestner KH (2005) The initiation of liver development is dependent on Foxa transcription factors. Nature 435: 944–947. 15959514 10.1038/nature03649

[7] Zhao RO, Watt AJ, Li JX, Luebke-Wheeler J, Morrisey EE, et al (2005) GATA6 is essential for embryonic development of the liver but dispensable for early heart formation. Molecular and Cellular Biology 25: 2622–2631. 15767668 10.1128/MCB.25.7.2622-2631.2005 PMC1061656

[8] Bort R, Signore M, Tremblay K, Martinez Barbera JP, Zaret KS (2006) Hex homeobox gene controls the transition of the endoderm to a pseudostratified, cell emergent epithelium for liver bud development. DevBiol 290: 44–56. 10.1016/j.ydbio.2005.11.006 16364283

[9] Sosa-Pineda B, Wigle JT, Oliver G (2000) Hepatocyte migration during liver development requires Prox1. Nature Genetics 25: 254–255. 10888866 10.1038/76996

[10] Wandzioch E, Zaret KS (2009) Dynamic signaling network for the specification of embryonic pancreas and liver progenitors. Science 324: 1707–1710. 10.1126/science.1174497 19556507 PMC2771431

[11] Jung J, Zheng M, Goldfarb M, Zaret KS (1999) Initiation of mammalian liver development from endoderm by fibroblast growth factors. Science 284: 1998–2003. 10373120 10.1126/science.284.5422.1998

[12] Rossi JM, Dunn NR, Hogan BL, Zaret KS (2001) Distinct mesodermal signals, including BMPs from the septum transversum mesenchyme, are required in combination for hepatogenesis from the endoderm. Genes Dev 15: 1998–2009. 11485993 10.1101/gad.904601 PMC312750

[13] Ober EA, Verkade H, Field HA, Stainier DY (2006) Mesodermal Wnt2b signalling positively regulates liver specification. Nature 442: 688–691. 16799568 10.1038/nature04888

[14] Stafford D, Prince VE (2002) Retinoic acid signaling is required for a critical early step in zebrafish pancreatic development. Current Biology 12: 1215–1220. 12176331 10.1016/s0960-9822(02)00929-6

[15] Chang C, Hu M, Zhu Z, Lo LJ, Chen J, et al (2011) Liver-Enriched Gene 1a and 1B Encode Novel Secretory Proteins Essential for Normal Liver Development in Zebrafish. PloS one 6: e22910 10.1371/journal.pone.0022910 21857963 PMC3153479

[16] Cheng W, Guo L, Zhang Z, Soo HM, Wen C, et al (2006) HNF factors form a network to regulate liver-enriched genes in zebrafish. Developmental biology 294: 482–496. 16631158 10.1016/j.ydbio.2006.03.018

[17] Enjapoori AK, Grant TR, Nicol SC, Lefevre CM, Nicholas KR, et al (2014) Monotreme lactation protein is highly expressed in monotreme milk and provides antimicrobial protection. Genome Biol Evol 6: 2754–2773. 10.1093/gbe/evu209 25245409 PMC4224336

[18] Lin TY, Chou CF, Chung HY, Chiang CY, Li CH, et al (2014) Hypoxia-Inducible Factor 2 Alpha Is Essential for Hepatic Outgrowth and Functions via the Regulation of leg1 Transcription in the Zebrafish Embryo. Plos One 9. 10.1371/journal.pone.0101980 PMC4084947 25000307

[19] Huang P, Xiao A, Zhou M, Zhu Z, Lin S, et al (2011) Heritable gene targeting in zebrafish using customized TALENs. Nat Biotechnol 29: 699–700. 10.1038/nbt.1939 21822242

[20] Rossi A, Kontarakis Z, Gerri C, Nolte H, Holper S, et al (2015) Genetic compensation induced by deleterious mutations but not gene knockdowns. Nature. 10.1038/nature14580 26168398

[21] Passeri MJ, Cinaroglu A, Gao C, Sadler KC (2009) Hepatic steatosis in response to acute alcohol exposure in zebrafish requires sterol regulatory element binding protein activation. Hepatology 49: 443–452. 10.1002/hep.22667 19127516 PMC2635426

[22] Kim BM, Rhee JS, Lee KW, Kim MJ, Shin KH, et al (2015) UV-B radiation-induced oxidative stress and p38 signaling pathway involvement in the benthic copepod Tigriopus japonicus. Comp Biochem Physiol C Toxicol Pharmacol 167: 15–23. 10.1016/j.cbpc.2014.08.003 25152408

[23] Walker SL, Ariga J, Mathias JR, Coothankandaswamy V, Xie X, et al (2012) Automated reporter quantification in vivo: high-throughput screening method for reporter-based assays in zebrafish. PLoS One 7: e29916 10.1371/journal.pone.0029916 22238673 PMC3251595

[24] Ellis JA, Cross AR, Jones OT (1989) Studies on the electron-transfer mechanism of the human neutrophil NADPH oxidase. Biochem J 262: 575–579. 2553003 10.1042/bj2620575 PMC1133307

[25] Stolk J, Hiltermann TJ, Dijkman JH, Verhoeven AJ (1994) Characteristics of the inhibition of NADPH oxidase activation in neutrophils by apocynin, a methoxy-substituted catechol. Am J Respir Cell Mol Biol 11: 95–102. 8018341 10.1165/ajrcmb.11.1.8018341

[26] Ober EA, Field HA, Stainier DYR (2003) From endoderm formation to liver and pancreas development in zebrafish. Mechanisms Of Development 120: 5–18. 12490292 10.1016/s0925-4773(02)00327-1

[27] Chen J, Ng SM, Chang C, Zhang Z, Bourdon JC, et al (2009) p53 isoform delta113p53 is a p53 target gene that antagonizes p53 apoptotic activity via BclxL activation in zebrafish. Genes Dev 23: 278–290. 10.1101/gad.1761609 19204115 PMC2648546

[28] Niu X, Gao C, Jan LL, Luo Y, Meng C, et al (2012) Sec13 safeguards the integrity of the endoplasmic reticulum and organogenesis of the digestive system in zebrafish. DevBiol 367: 197–207. 10.1016/j.ydbio.2012.05.004 22609279

[29] Deutsch G, Jung J, Zheng M, Lora J, Zaret KS (2001) A bipotential precursor population for pancreas and liver within the embryonic endoderm. Development 128: 871–881. 11222142 10.1242/dev.128.6.871

[30] Wallace KN, Pack M (2003) Unique and conserved aspects of gut development in zebrafish. Dev Biol 255: 12–29. 12618131 10.1016/s0012-1606(02)00034-9

[31] Huang H, Ruan H, Aw MY, Hussain A, Guo L, et al (2008) Mypt1-mediated spatial positioning of Bmp2-producing cells is essential for liver organogenesis. Development 135: 3209–3218. 10.1242/dev.024406 18776143 PMC5574253

[32] Shin D, Shin CH, Tucker J, Ober EA, Rentzsch F, et al (2007) Bmp and Fgf signaling are essential for liver specification in zebrafish. Development 134: 2041–2050. 17507405 10.1242/dev.000281

[33] Negishi T, Nagai Y, Asaoka Y, Ohno M, Namae M, et al (2010) Retinoic acid signaling positively regulates liver specification by inducing wnt2bb gene expression in medaka. Hepatology 51: 1037–1045. 10.1002/hep.23387 19957374

[34] Wilkinson RN, Pouget C, Gering M, Russell AJ, Davies SG, et al (2009) Hedgehog and Bmp polarize hematopoietic stem cell emergence in the zebrafish dorsal aorta. Dev Cell 16: 909–916. 10.1016/j.devcel.2009.04.014 19531361 PMC3210643

[35] Rian H, Krens SFG, Spaink HP, Snaar-Jagalska BE (2013) Generation of Constitutive Active ERK Mutants as Tools for Cancer Research in Zebrafish. ISRN Cell Biology 2013: 1–11.

[36] Poulain M, Furthauer M, Thisse B, Thisse C, Lepage T (2006) Zebrafish endoderm formation is regulated by combinatorial Nodal, FGF and BMP signalling. Development 133: 2189–2200. 16672336 10.1242/dev.02387

[37] Loew R, Heinz N, Hampf M, Bujard H, Gossen M (2010) Improved Tet-responsive promoters with minimized background expression. BMC Biotechnol 10: 81 10.1186/1472-6750-10-81 21106052 PMC3002914

[38] Gossen M, Freundlieb S, Bender G, Muller G, Hillen W, et al (1995) Transcriptional activation by tetracyclines in mammalian cells. Science 268: 1766–1769. 7792603 10.1126/science.7792603

[39] Apweiler R, Hermjakob H, Sharon N (1999) On the frequency of protein glycosylation, as deduced from analysis of the SWISS-PROT database. Biochimica Et Biophysica Acta-General Subjects 1473: 4–8. 10.1016/s0304-4165(99)00165-8 10580125

[40] Moremen KW, Tiemeyer M, Nairn AV (2012) Vertebrate protein glycosylation: diversity, synthesis and function. Nat Rev Mol Cell Biol 13: 448–462. 10.1038/nrm3383 22722607 PMC3934011

[41] Gonzalez J, Takao T, Hori H, Besada V, Rodriguez R, et al (1992) A Method for Determination Of N-Glycosylation Sites In Glycoproteins by Collision-Induced Dissociation Analysis In Fast-Atom-Bombardment Mass-Spectrometry—Identification Of the Positions Of Carbohydrate-Linked Asparagine In Recombinant Alpha-Amylase by Treatment with Peptide-N-Glycosidase-F In O-18-Labeled Water. Analytical Biochemistry 205: 151–158. 1443554 10.1016/0003-2697(92)90592-u

[42] Koutsioulis D, Landry D, Guthrie EP (2008) Novel endo-alpha-N-acetylgalactosaminidases with broader substrate specificity. Glycobiology 18: 799–805. 10.1093/glycob/cwn069 18635885 PMC2553423

[43] R. Gupta EJaSB (2004) Prediction of N-glycosylation sites in human proteins.

[44] Molinari M (2007) N-glycan structure dictates extension of protein folding or onset of disposal. Nat Chem Biol 3: 313–320. 17510649 10.1038/nchembio880

[45] Haugg M, Schein CH (1992) The DNA sequences of the human and hamster secretory ribonucleases determined with the polymerase chain reaction (PCR). Nucleic Acids Res 20: 612 1741299 10.1093/nar/20.3.612 PMC310435

[46] Linstedt AD, Hauri HP (1993) Giantin, a novel conserved Golgi membrane protein containing a cytoplasmic domain of at least 350 kDa. Mol Biol Cell 4: 679–693. 7691276 10.1091/mbc.4.7.679 PMC300978

[47] Touret N, Paroutis P, Terebiznik M, Harrison RE, Trombetta S, et al (2005) Quantitative and dynamic assessment of the contribution of the ER to phagosome formation. Cell 123: 157–170. 16213220 10.1016/j.cell.2005.08.018

[48] Lee Y, Grill S, Sanchez A, Murphy-Ryan M, Poss KD (2005) Fgf signaling instructs position-dependent growth rate during zebrafish fin regeneration. Development 132: 5173–5183. 16251209 10.1242/dev.02101

[49] Rossi A, Kontarakis Z, Gerri C, Nolte H, Holper S, et al (2015) Genetic compensation induced by deleterious mutations but not gene knockdowns. Nature 524: 230–233. 10.1038/nature14580 26168398

[50] Gilbert SF (2012) Ecological developmental biology: environmental signals for normal animal development. Evol Dev 14: 20–28. 10.1111/j.1525-142X.2011.00519.x 23016971

[51] Goldfarb M (2001) Signaling by fibroblast growth factors: the inside story. Sci STKE 2001: pe37 11687709 10.1126/stke.2001.106.pe37 PMC3208904

[52] Miyazono K, Kamiya Y, Morikawa M (2010) Bone morphogenetic protein receptors and signal transduction. J Biochem 147: 35–51. 10.1093/jb/mvp148 19762341

[53] Rao TP, Kuhl M (2010) An updated overview on Wnt signaling pathways: a prelude for more. Circ Res 106: 1798–1806. 10.1161/CIRCRESAHA.110.219840 20576942

[54] Chang C, Hu M, Zhu Z, Lo LJ, Chen J, et al (2011) liver-enriched gene 1a and 1b encode novel secretory proteins essential for normal liver development in zebrafish. PLoSOne 6: e22910. 10.1371/journal.pone.0022910 PMC3153479 21857963

[55] Yang SL, Aw SS, Chang C, Korzh S, Korzh V, et al (2011) Depletion of Bhmt elevates sonic hedgehog transcript level and increases beta-cell number in zebrafish. Endocrinology 152: 4706–4717. 10.1210/en.2011-1306 21952238

[56] Anichtchik O, Diekmann H, Fleming A, Roach A, Goldsmith P, et al (2008) Loss of PINK1 function affects development and results in neurodegeneration in zebrafish. J Neurosci 28: 8199–8207. 10.1523/JNEUROSCI.0979-08.2008 18701682 PMC6670558

[57] Krzywinski M, Altman N (2013) Points of significance: error bars. Nat Methods 10: 921–922. 10.1038/nmeth.2659 24161969

[58] Krzywinski M, Altman N (2014) Points of Significance: Visualizing samples with box plots. Nat Meth 11: 119–120. 10.1038/nmeth.2813 24645192

[59] Ginestet C (2011) ggplot2: Elegant Graphics for Data Analysis. Journal Of the Royal Statistical Society Series a-Statistics In Society 174: 245–245.

