# Supplementary material for: Liver-Enriched Gene 1, a Glycosylated Secretory Protein, Binds to FGFR and Mediates an Anti-stress Pathway to Protect Liver Development in Zebrafish
Source: PLoS Genet. 2016 Feb 22;12(2):e1005881. doi: 10.1371/journal.pgen.1005881 (PMC4764323; doi:10.1371/journal.pgen.1005881)
Supplement: S1 Table — (DOCX) [file pgen.1005881.s001.docx]

| **S1 Table. Primers used to generate *leg1* mutant.** | |
| --- | --- |
| *leg1a^N70A^* fwd | CAAAATGCTGATCGCCGCTACTAATGC |
| *leg1a^N70A^* rev | GCATTAGTAGCGGCGATCAGCATTTTG |
| *leg1b^N70A^* fwd | GAAAATGGTGATCGCCGCTACTAATGC |
| *leg1b^N70A^* rev | GCATTAGTAGCGGCGATCACCATTTTC |
| *leg1a^N298A^* fwd | GCACTGAAGAGGCACACACACTCTAC |
| *leg1a^N298A^* rev | TCTCACACACACGGAGAAGTCACGA |
