# Supplementary material for: Liver-Enriched Gene 1, a Glycosylated Secretory Protein, Binds to FGFR and Mediates an Anti-stress Pathway to Protect Liver Development in Zebrafish
Source: PLoS Genet. 2016 Feb 22;12(2):e1005881. doi: 10.1371/journal.pgen.1005881 (PMC4764323; doi:10.1371/journal.pgen.1005881)
Supplement: S2 Table — (DOCX) [file pgen.1005881.s002.docx]

| S2 Table. Primers for qPCR | |
| --- | --- |
| *leg1* Fw | GATGTGGGCTGCCGAGGAGGAG |
| *leg1* Rv | TCTGCGATGTTGGGTGGACTGT |
| *leg1b* Fw | GCTCAGCTGGATGAACAGCA |
| *leg1b* Rv | ggtgagagtgtgtcagcag |
| *atf6* Fw | CTggAggCgCTggTgAAAAgTg |
| *atf6* Rv | CCggACgggAgATgggAACA |
| *bip* Fw | ggTCggCggCTCCACTCgTAT |
| *bip* Rv | AgCCggTTCTggTCgTTggTAATg |
| *chop* Fw | ggAgCCCgAgTTTTTggATgTTTT |
| *chop* Rv | AgTgCgCCgCCTCgTTTTCTT |
| *grp94* Fw | TCCCgCACCgATgACgA |
| *grp94* Rv | gTAgAAgCCCACACCgAACTgAC; |
| *ire1a* Fw | ATggCgTggggAgTgTgC |
| *ire1a* Rv | gTATTCTgTgCggCCAAggTAAA |
| *perk* Fw | CCgCggggCAACAgAgT |
| *perk* Rv | ggTggCAgCgATACAgAAgAAgAT |
| *actin* Fw | ATGGATGAGGAAATCGCTGCC |
| *actin* Rv | CTCCCTGATGTCTGGGTCGTC |
