# Supplementary figures and images for: Liver-Enriched Gene 1, a Glycosylated Secretory Protein, Binds to FGFR and Mediates an Anti-stress Pathway to Protect Liver Development in Zebrafish
Source: PLoS Genet. 2016 Feb 22;12(2):e1005881. doi: 10.1371/journal.pgen.1005881 (PMC4764323; doi:10.1371/journal.pgen.1005881)

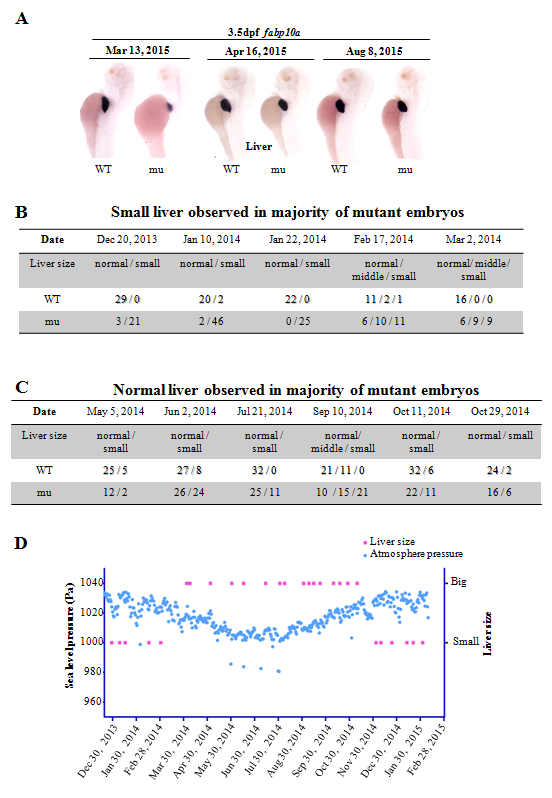

Supplement: S1 Fig — (A) Representative images of embryos after WISH using the fabp10a probe corresponding to the result shown in Fig 1F. (B and C) Among the 32 cases shown in Fig 1G, the number of embryos exhibiting a small versus normal liver in 5 cases recorded in cold seasons (B) and 6 cases recorded in warm/hot seasons (C) were shown. (D) Plotting the liver sizes (majority normal or majority small) against daily atmospheric pressure in Hangzhou recorded during 30/12/2013 and 20/02/2015 (obtained from http://www.wunderground.com/). (TIFF) [file pgen.1005881.s003.TIFF]

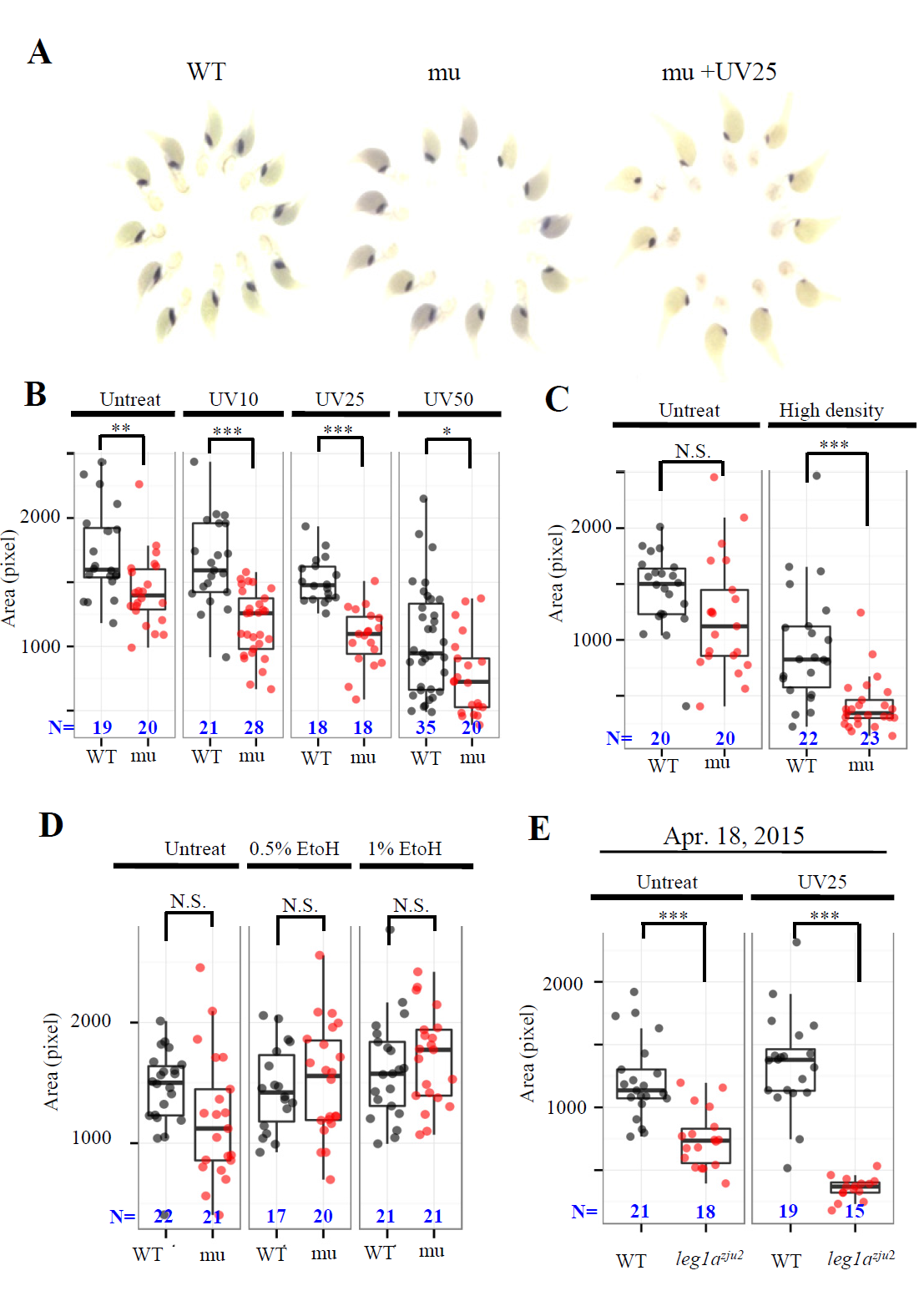

Supplement: S2 Fig — (A) Images showing an example of determining the liver size by WISH using the fabp10a probe. WT: wild type; mu: leg1azju1 mutant; mu+UV25: leg1azju1 mutant treated with UV25. (B) WT and maternal-zygotic leg1azju1(mu) embryos were treated with 1 mJ/cm2 (UV10), 2.5 mJ/cm2(UV25) and 5 mJ/cm2 (UV50) UV at 24 hpf and grew to 3.5 dpf for WISH analysis of liver development. (C) Comparison of liver sizes between the WT and maternal-zygotic leg1azju1 (mu) embryos growing in a high density condition (200 embryos per 10-cm diameter Petri dish). (D) Growing the maternal-zygotic leg1azju1 (mu) embryos in the egg water containing 0.5% or 1% ethanol did not cause a small liver phenotype. (E) Upon UV25 treatment the maternal-zygotic leg1azju2 embryos also exhibited a small liver phenotype at 3.5 dpf. *, p<0.05, **, p<0.01, ***, p<0.001, N.S., no significance. (TIFF) [file pgen.1005881.s004.TIFF]

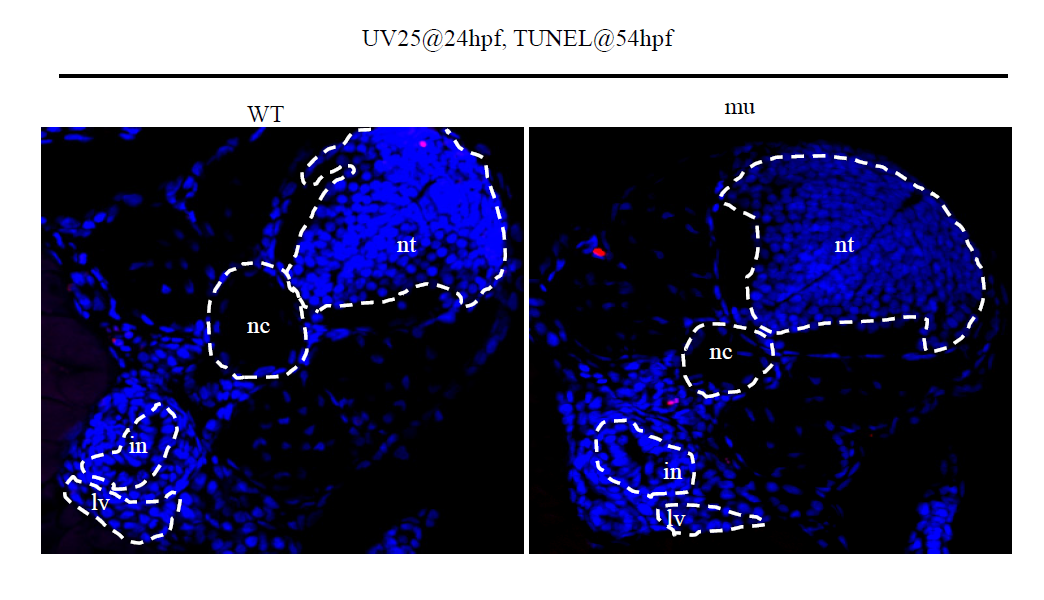

Supplement: S3 Fig — Images of TUNEL assay in the 54-hpf WT and maternal-zygotic leg1azju1 embryos (mu) after UV25 treatment at 24 hpf. No abnormal apoptotic activity was observed near the endodermal region including liver (lv) and intestine (in) in the maternal-zygotic leg1azju1 embryos (mu) compared to the WT. 12 sections from six embryos for each genotype were examined. nc, notochord, nt, neural tube. (TIFF) [file pgen.1005881.s005.TIFF]

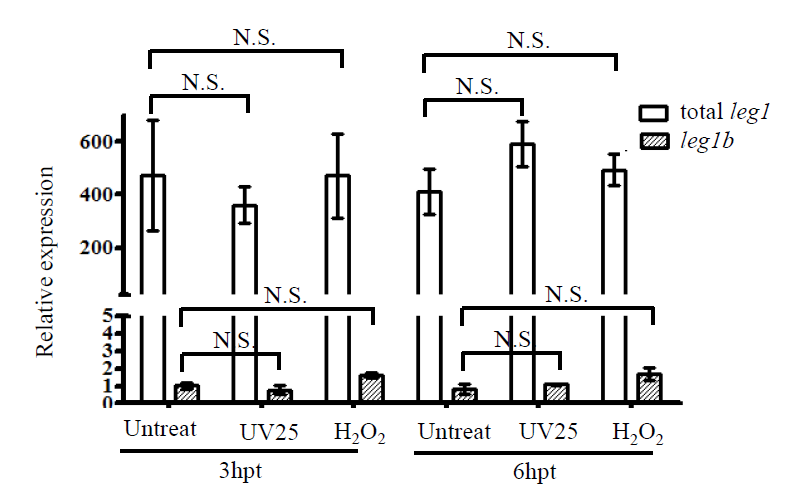

Supplement: S4 Fig — The 24-hpf WT embryos were treated with UV25 or 0.5 mM H2O2. Total leg1 or leg1b RNA level were measured by quantitative PCR (qPCR) using leg1a and leg1b common primers or leg1b specific primers, respectively. Error bar stands for the standard error. N.S., no significance. (TIFF) [file pgen.1005881.s006.TIFF]

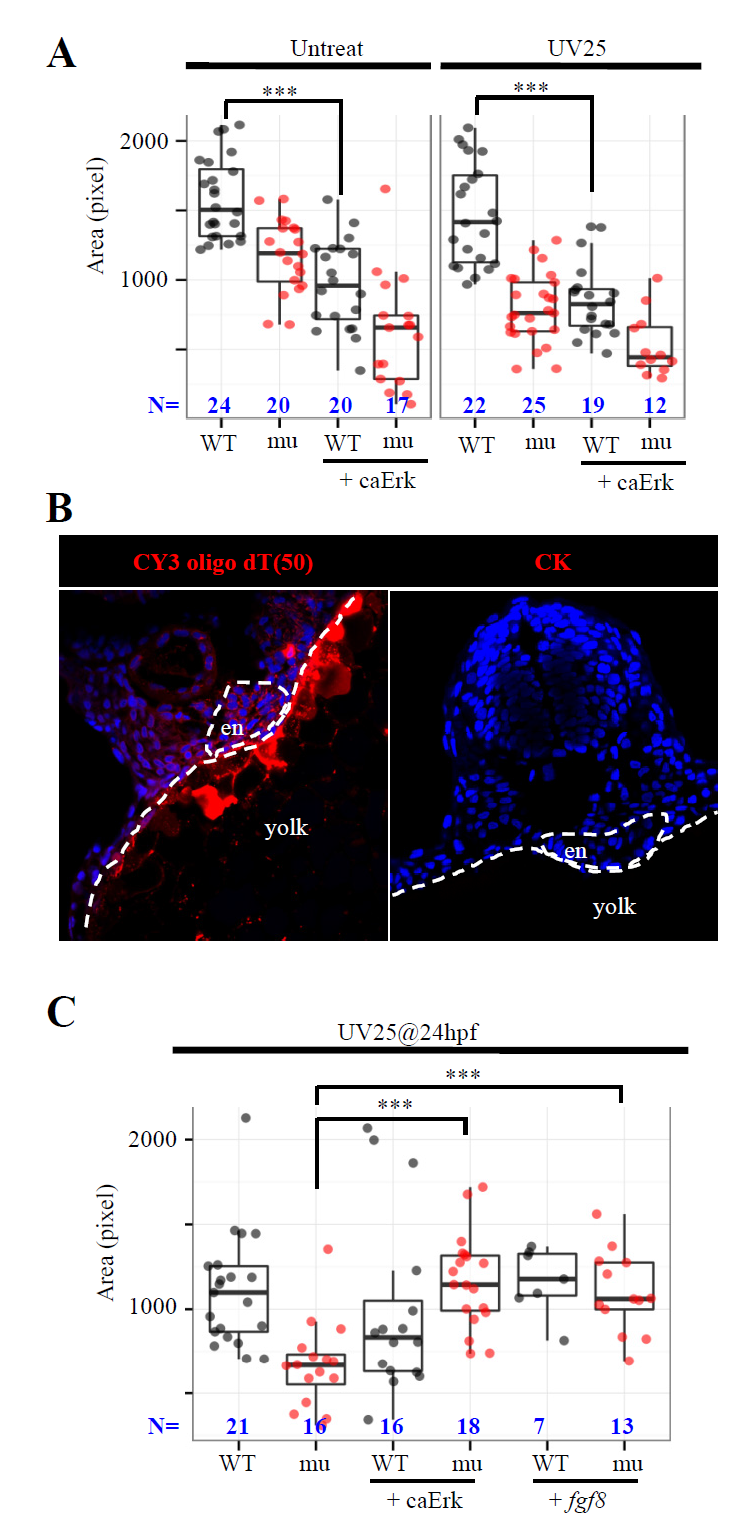

Supplement: S5 Fig — (A) Injection of constitutively active form of Erk (caErk) mRNA into one-cell stage embryos impaired liver development both in WT and leg1azju1 mutant when examined with the fabp10a probe at 3.5 dpf. (B) 200 pg Cy3 labeled oligo-dT(50) was injected into the yolk at 22 hpf, and the Cy3 signal was checked at 27 hpf. CK, oligo-dT(50) uninjected control. (C) Embryos were injected with caErk or fgf8 mRNA into the yolk at 22 hpf and were then treated with UV25 at 24 hpf. The liver development in the treated embryos at 3.5 dpf was examined with the fabp10a probe at 3.5 dpf. (TIFF) [file pgen.1005881.s007.TIFF]

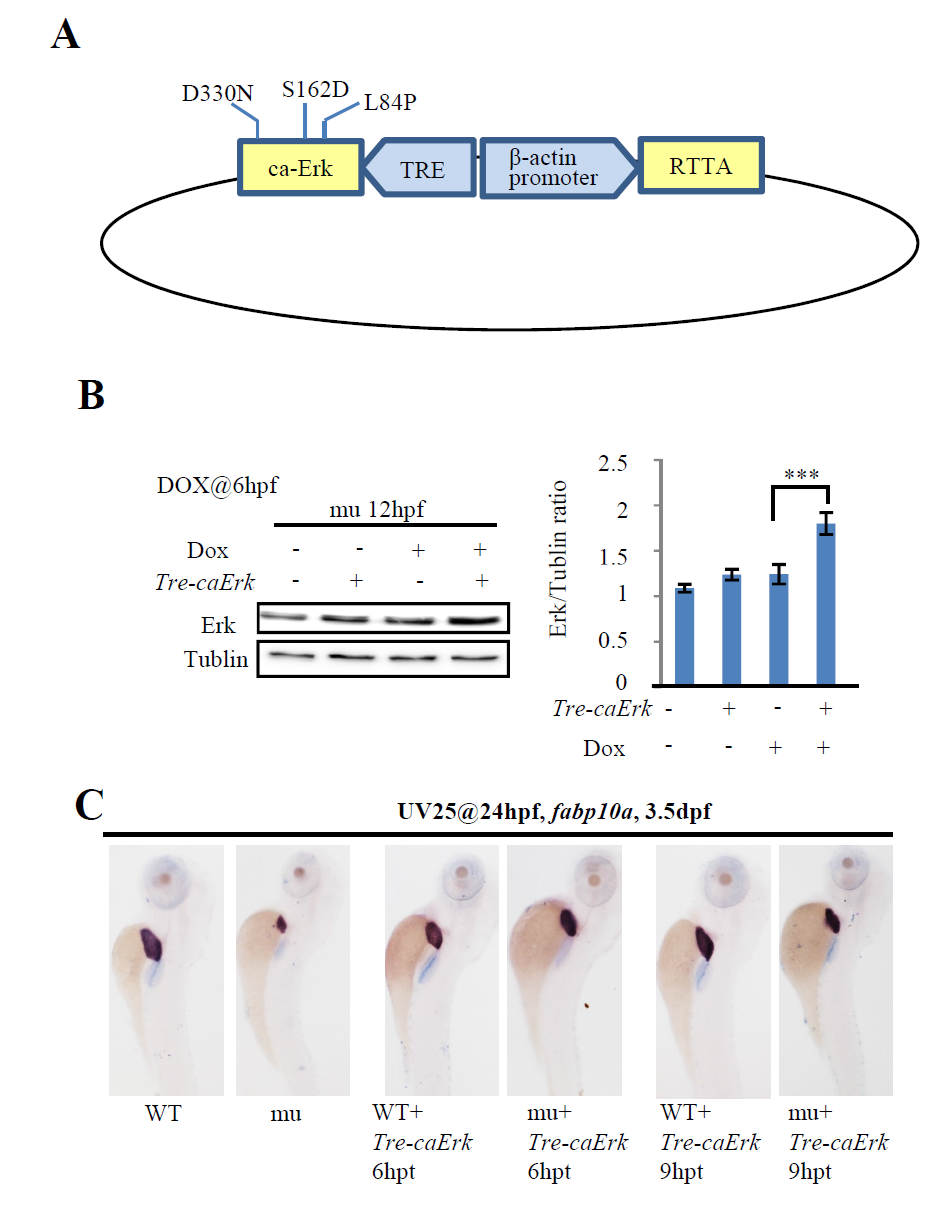

Supplement: S6 Fig — (A) Schematic drawing showing the structure of the TRE-caErk plasmid. RTTA expression was driven by the β-actin gene promoter. Dox binds to RTTA and the Dox-RTTA complex binds to the TRE promoter to drive the expression of caErk. (B) 10 pgTre-caErk plasmid DNA was injected into one-cell stage maternal-zygotic leg1azju1 embryos (mu). These embryos were treated with Dox at 6 hpf and total protein was harvested at 12 hpf. The protein samples were subjected to western blot analysis. The total Erk versus Tubulin ratios were shown on the right. Dox, doxycycline. Error bar stands for the standard error. ***, p<0.001. Western blot was repeated three times. (C) Images of representative 3.5-dpf embryos after WISH using the fabp10a probe. Embryos was first injected with Tre-caErk plasmid at one-cell stage, then treated with UV25 at 24 hpf and followed by Dox treatment for 6 hours (6 hpt) or 9 hours (9 hpt). After Dox treatment, embryos were transferred to the normal egg water to grow to 3.5 dpf for WISH (n = 20). (TIFF) [file pgen.1005881.s008.TIFF]

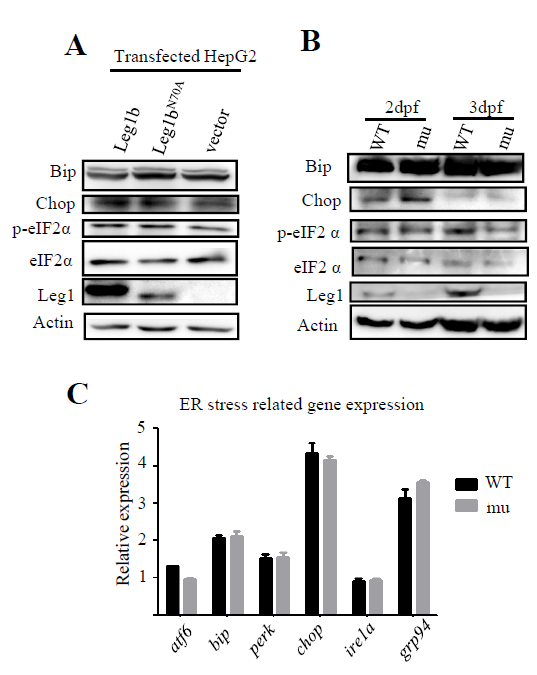

Supplement: S7 Fig — (A) HepG2 cells were transfected with the leg1b, leg1bN70A, and the vector plasmid DNA. Total protein was extracted 30 hours post transfection and subjected to western analysis of Bip, Chop, phosphorylated eIF2α (p-eIF2α), and total eIF2α. These ER-stress response markers were not activated by the hypoglycosylated Leg1bN70A. Vector, the PCS2+ vector transfected cell. (B) Western blot analysis of Bip, Chop, phosphorylated eIF2α (p-eIF2α), and total eIF2α in the WT and maternal-zygotic leg1azju1 mutant embryos at 2 dpf and 3 dpf. (C) qPCR analysis of the transcript levels of ER-stress response markers including atf6, bip, perk, chop, ire1a, and grp94 in 3 dpf WT and maternal-zygotic leg1azju1 mutant embryos. Error bar stands for the standard error. Primers for analyzing these ER stress marker was as previously reported (S2 Table). Western blot was repeated three times each for A and B. (TIFF) [file pgen.1005881.s009.TIFF]

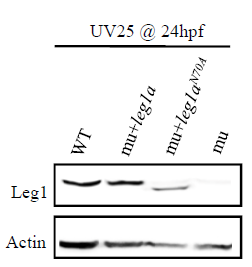

Supplement: S8 Fig — Corresponding to Fig 7A and 7B. Western blot analysis of Leg1a or Leg1aN70A protein in 3 dpf old maternal-zygotic leg1azju1 mutant embryos injected with leg1a (mu+1a) orleg1aN70A(mu+1aN70A) mRNA at the one-cell stage. Protein samples from the WT and maternal-zygotic leg1azju1 mutant (mu) embryos were used as controls. Western blot was repeated three times. (TIFF) [file pgen.1005881.s010.TIFF]

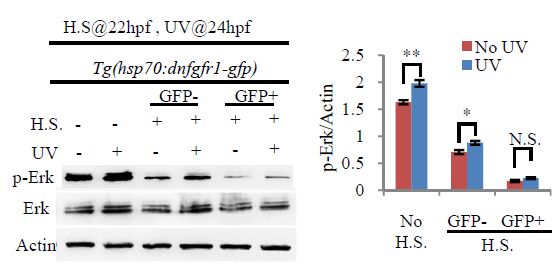

Supplement: S9 Fig — Tg(hsp70:dnfgfr1-gfp) embryos were heatshocked at 22 hpf to induce the expression of dominant negative FGFR1 (dn-Fgfr1). GFP signal was used to distinguish the dn-Fgfr-expressed (GFP+) and non-dn-Fgfr-expressed (GFP-) embryos. Embryos were treated with UV25 at 24 hpf, and total protein was extracted from embryos at 30 hpf (6 h post treatment) and was subjected to western analysis of the level of p-Erk. Tublin was used as a loading control. H.S., heatshock. *, p<0.05, **, p<0.01, N.S., no significance. Western blot was repeated two times. (TIFF) [file pgen.1005881.s011.TIFF]

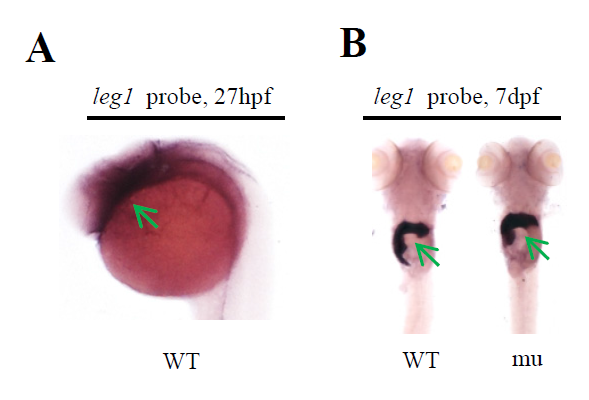

Supplement: S10 Fig — n = 25. (TIFF) [file pgen.1005881.s012.TIFF]

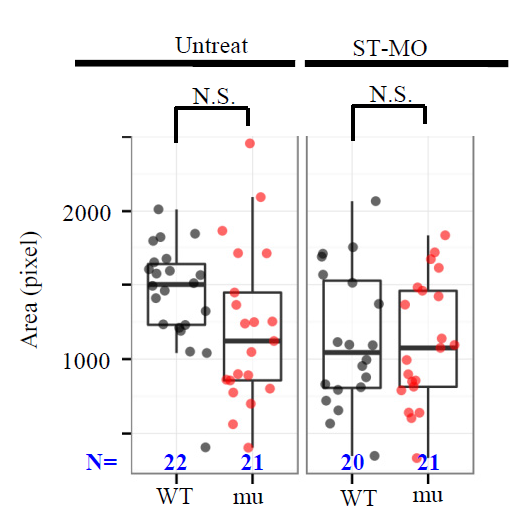

Supplement: S11 Fig — One nanolitre of 0.5 mM standard control mopholino (ST-MO) was injected into one-cell stage embryos. The liver development was examined at 3.5 dpf using the fabp10a probe. N.S., no significance. (TIFF) [file pgen.1005881.s013.TIFF]

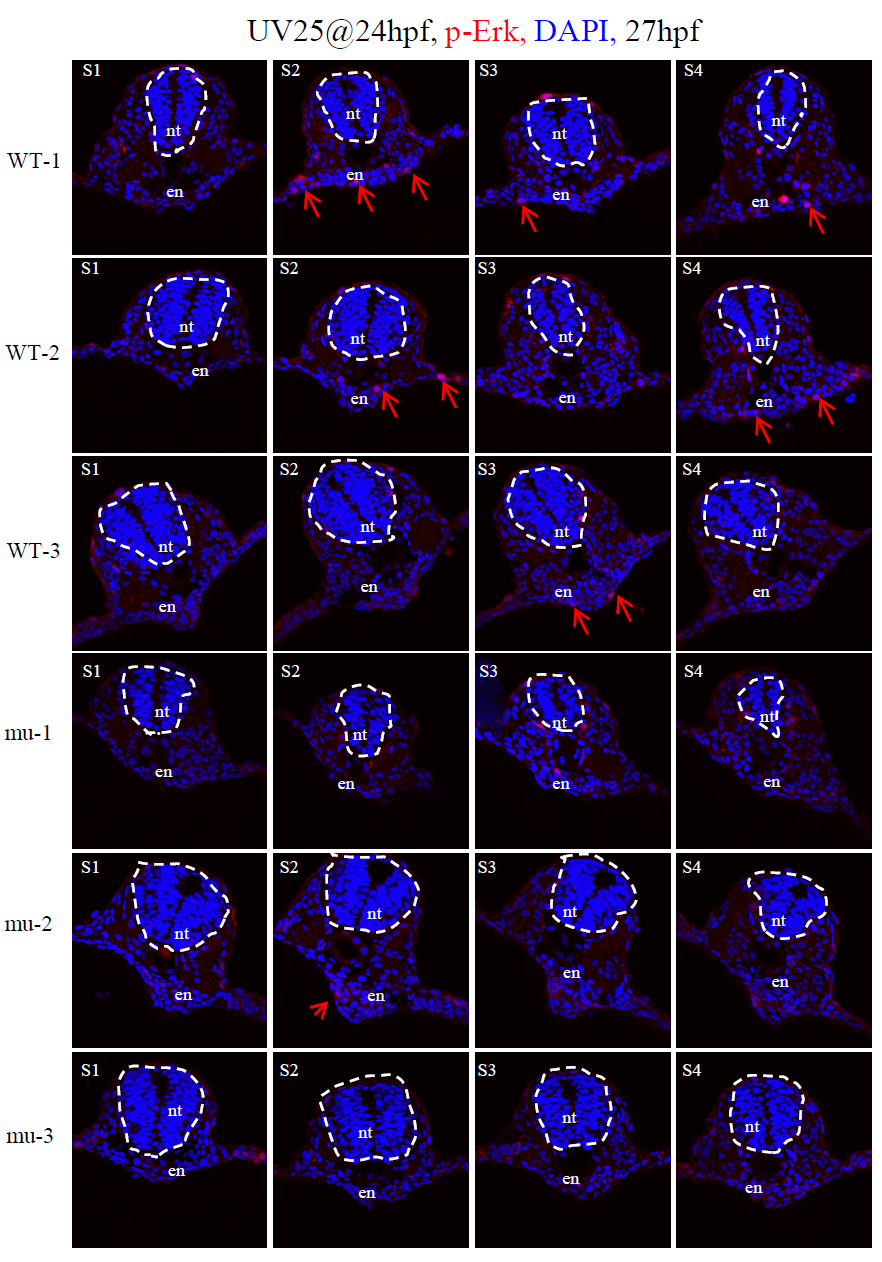

Supplement: S12 Fig — Serial cryosections (S1 to S4) from three WT embryos (WT-1, WT-2 and WT-3) and three mutant embryos (mu-1, mu-2 and mu-3) treated with UV25 at 24 hpf were shown. DAPI was used to stain the nuclei. (TIFF) [file pgen.1005881.s014.TIFF]
